# DNA methylation and *cis*-regulation of gene expression by prostate cancer risk SNPs

**DOI:** 10.1371/journal.pgen.1008667

**Published:** 2020-03-30

**Authors:** James Y. Dai, Xiaoyu Wang, Bo Wang, Wei Sun, Kristina M. Jordahl, Suzanne Kolb, Yaw A. Nyame, Jonathan L. Wright, Elaine A. Ostrander, Ziding Feng, Janet L. Stanford

**Affiliations:** 1 Division of Public Health Sciences, Fred Hutchison Cancer Research Center, Seattle, Washington, United States of America; 2 Department of Biostatistics, University of Washington School of Public Health, Seattle, Washington, United States of America; 3 Department of Laboratory Medicine, Shanghai Children’s Medical Center, Shanghai Jiao Tong University School of Medicine, Shanghai, China; 4 Department of Urology, University of Washington School of Medicine, Seattle, Washington, United States of America; 5 Cancer Genetics and Comparative Genomics Branch, National Human Genome Research Institute, NIH, Bethesda, Maryland, United States of America; 6 Department of Epidemiology, University of Washington School of Public Health, Seattle, Washington, United States of America; Case Western Reserve University, UNITED STATES

## Abstract

Genome-wide association studies have identified more than 100 SNPs that increase the risk of prostate cancer (PrCa). We identify and compare expression quantitative trait loci (eQTLs) and CpG methylation quantitative trait loci (meQTLs) among 147 established PrCa risk SNPs in primary prostate tumors (n = 355 from a Seattle-based study and n = 495 from The Cancer Genome Atlas, TCGA) and tumor-adjacent, histologically benign samples (n = 471 from a Mayo Clinic study). The role of DNA methylation in eQTL regulation of gene expression was investigated by data triangulation using several causal inference approaches, including a proposed adaptation of the Causal Inference Test (*CIT*) for causal direction. Comparing eQTLs between tumors and benign samples, we show that 98 of the 147 risk SNPs were identified as eQTLs in the tumor-adjacent benign samples, and almost all 34 eQTL identified in tumor sets were also eQTLs in the benign samples. Three lines of results support the causal role of DNA methylation. First, nearly 100 of the 147 risk SNPs were identified as meQTLs in one tumor set, and almost all eQTLs in tumors were meQTLs. Second, the loss of eQTLs in tumors relative to benign samples was associated with altered DNA methylation. Third, among risk SNPs identified as both eQTLs and meQTLs, mediation analyses suggest that over two-thirds have evidence of a causal role for DNA methylation, mostly mediating genetic influence on gene expression. In summary, we provide a comprehensive catalog of eQTLs, meQTLs and putative cancer genes for known PrCa risk SNPs. We observe that a substantial portion of germline eQTL regulatory mechanisms are maintained in the tumor development, despite somatic alterations in tumor genome. Finally, our mediation analyses illuminate the likely intermediary role of CpG methylation in eQTL regulation of gene expression.

## Author summary

We conduct rigorous eQTL and meQTL mapping for the 147 confirmed PrCa risk SNPs using comprehensive genomic data in primary prostate tumors (TCGA and FHCRC) and tumor-adjacent benign samples (Mayo Clinic). The goal is to explore the biological mechanisms of how SNPs predispose to PrCa risk, and to investigate the causal role of DNA methylation in genetic regulation of gene expression. To our knowledge this is the first eQTL study for PrCa risk SNPs that includes the comparison between tumors and benign samples, as well as studying DNA methylation. We use several causal inference approaches, including a proposed adaptation of the Causal Inference Test (*CIT*) to decipher the direction of causality. We provide a comprehensive catalog of eQTLs, meQTLs and putative cancer genes for known PrCa risk SNPs, which shows eQTL regulatory mechanisms largely maintained in prostate tumors, and our mediation analyses shed light on the intermediary role of CpG methylation in eQTL regulation of gene expression.

## Introduction

**Fig 1:**
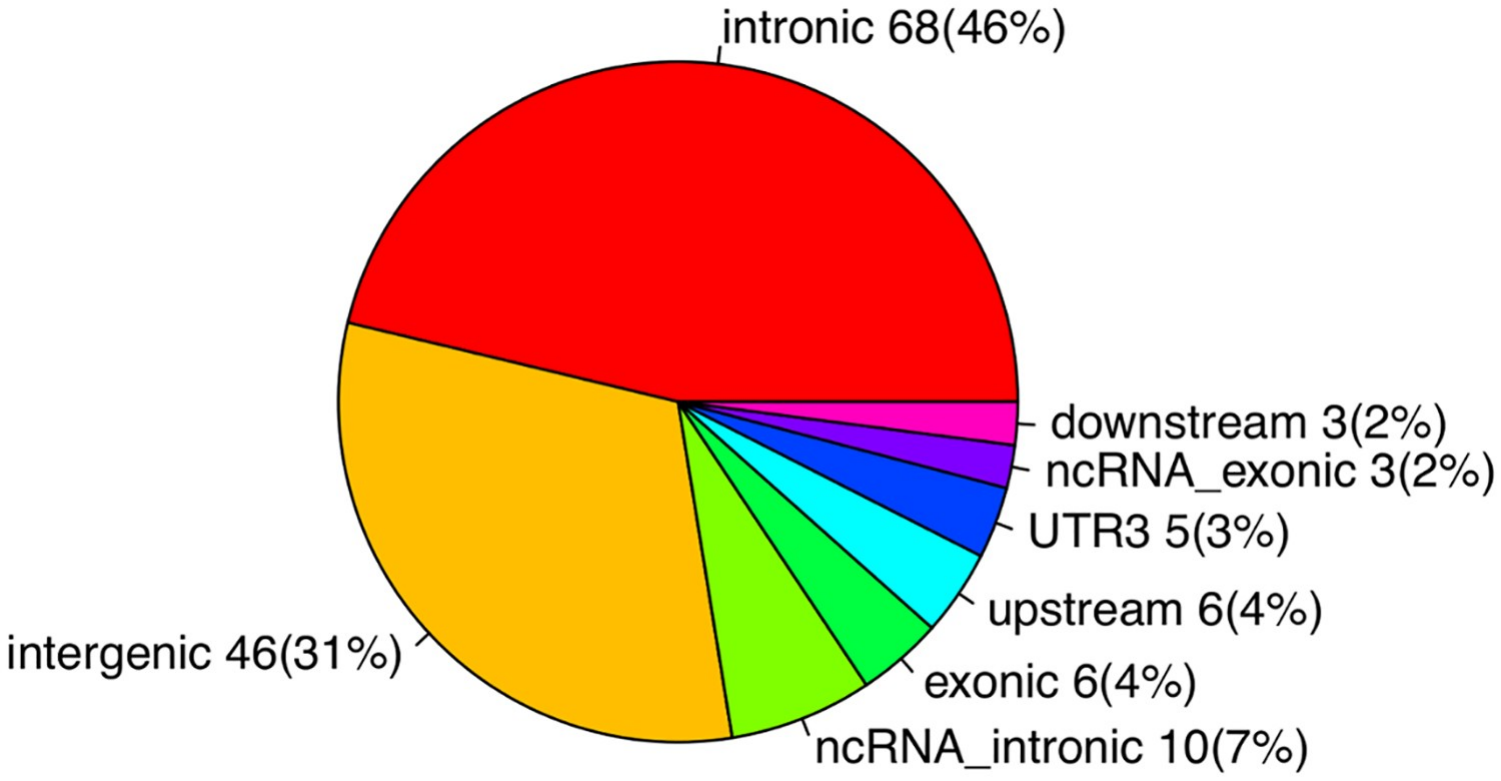
The genomic locations of the 147 prostate cancer risk SNPs.

Prostate cancer (PrCa) is the most common noncutaneous cancer among men in the Western world [1], yet few risk factors have been identified [2]. Twin and familial studies have long established that genetics is a major component of PrCa etiology [3–8]. Tremendous progress has been made by genome-wide association studies (GWAS) to identify genetic loci predisposing to PrCa, and more than 150 PrCa susceptibility SNPs have been identified [9–29]. The most recent and the largest GWAS to date assembled 79,194 cases and 61,112 controls of European ancestry from 59 studies for genotyping on a custom high-density genotyping array (the OncoArray), identified 62 novel loci associated with overall PrCa risk [29]. The polygenic risk score using the more commonly occurring 147 PrCa risk SNPs (S1 Table) captured 28.8% of the familial relative risk, which may provide a useful tool to identify men at higher risk for PrCa. Despite the remarkable progress in identifying PrCa risk SNPs, functional interpretation of risk SNPs presents a huge challenge. As Fig 1 shows, 141 out of the 147 PrCa risk SNPs (96%) are located in noncoding regions of the human genome, and five of six risk SNPs located in coding regions are nonsynonymous. The biological mechanisms of these SNPs influencing the risk of PrCa remain largely unknown.

A common strategy to interpret functional activity of GWAS-identified risk SNPs is to investigate whether they affect gene expression [30–33]. Such genetic determinants are referred to as expression quantitative trait loci (eQTLs), influencing mostly local genes in nearby genomic regions (local eQTLs, or *cis-*eQTLs for convenience, without requiring evidence of allelic effects at each locus). Large consortia such as the Genotype Tissue Expression project (GTEx) now provide genome-wide eQTL mapping from normal whole blood samples and multiple organs [34,35]. Evidence is abundant that trait-associated SNPs are more likely to be eQTLs [36], and eQTLs are pervasive in the human genome. A substantial number of eQTLs in the human genome are tissue-specific, and sample size is a major determinant of the number of eQTLs that can be detected at genome-wide significance [35]. Yet the number of normal prostate samples in GTEx is limited due to the difficulty of obtaining normal prostate samples from donors. A large-scale prostate-specific eQTL analysis from the Mayo Clinic was conducted in 2015 using 471 adjacent histologically benign prostate tissue samples from prostate cancer patients [37], reporting that nearly half of the known PrCa risk loci/regions may harbor *cis-*eQTLs. As eQTLs are often dependent on tissue type and developmental stage, it is anticipated that eQTLs will differ between primary prostate tumor tissue and adjacent benign tissue from the same prostate, though there has not been a systematic comparison of eQTLs and associated genes (referred to as eGenes hereafter) for the most recent set of GWAS loci between primary prostate cancer samples and tumor-adjacent benign prostate samples. The differences in eQTL associations may reflect molecular alterations that occur during carcinogenesis. An investigation of eQTLs among 12 known PrCa risk loci in 2012 identified four *cis*-QTLs in benign tissues and these associations tended to be more attenuated in tumors [38]. Another eQTL study in 2015 examined 39 PrCa risk loci and differences of associations between tumor and adjacent benign samples [39].

While a great deal of progress has been made in identifying eQTLs in the human genome, epigenetic mechanisms for regulating gene expression, e.g., DNA methylation, histone modification, and chromatin accessibility, are less understood [40,41,42]. In particular, DNA methylation at CpG sites is an essential epigenetic mark that links to cellular differentiation and tissue development. Aberrant DNA methylation has been long recognized to be associated with human diseases, including cancers [43]. This is particularly of interest to prostate cancer, as DNA methylation plays a critical role in developing prostate cancer, and epigenome continues to evolve throughout the life history of prostate cancer, possibly associated with cancer outcome [44]. Genetic polymorphisms such as SNPs have been shown to contribute to variations of DNA methylation in blood, brain, and adipose tissue and are referred to as methylation-QTLs (meQTLs) [45–49]. Interestingly, accumulating evidence from peripheral blood samples suggests that eQTLs co-localize with meQTLs in the genome [41,50–52], and genetic control of DNA methylation and gene expression may have a shared, causal component [53]. Dissecting the direction of causality presents challenge to statistical analysis: DNA methylation could be associated with SNPs independent of an effect on gene expression, or actively mediate the eQTL effect on gene expression, or play a passive role in gene regulation as a downstream event. The Mendelian randomization approach has been used in this context, which assumes that the genetic effect on an outcome goes through the intermediary in its entirety and therefore cannot test against the independence relationship [54]. The Causal Inference Test (*CIT*) can test the causal direction in principle, using the conditional independence relationship of three variables in a causal pathway and simultaneously assessing multiple causal parameters in the pathway models [55]. However in practice it can produce significance for both mediation and reverse causation, rendering results uninterpretable [55]. To date, no meQTL mapping study has been reported on prostate tumor samples or histologically benign prostate samples, nor has any study disentangled the relationships between eQTLs, DNA methylation and gene expression in prostate tissue samples.

In this work, we perform a comprehensive eQTL and meQTL analysis for 147 PrCa risk SNPs using genomic data from two large sets of primary prostate tumor samples (Fred Hutchinson Cancer Research Center, FH, n = 355; and The Cancer Genome Atlas, TCGA, n = 495) and a large set of histologically benign prostate samples from cancer patients (Mayo Clinic, n = 471). Focusing on established PrCa risk SNPs, the goal of this analysis is three-fold: to identify and compare eQTLs in tumor-adjacent benign samples and primary prostate tumors, to identify meQTLs in tumor-adjacent benign samples, and to investigate through data triangulation the causal role of DNA methylation in genetic regulation of gene expression. Genes found to be under regulation of the PrCa risk SNPs will be characterized by Ingenuity Pathway Analysis (IPA). To interpret eQTLs in the context of cancer development, we also analyze data from 50 pairs of tumor and tumor-adjacent, histologically benign samples from TCGA.

## Results

### Overview of samples and datasets

**Fig 2:**
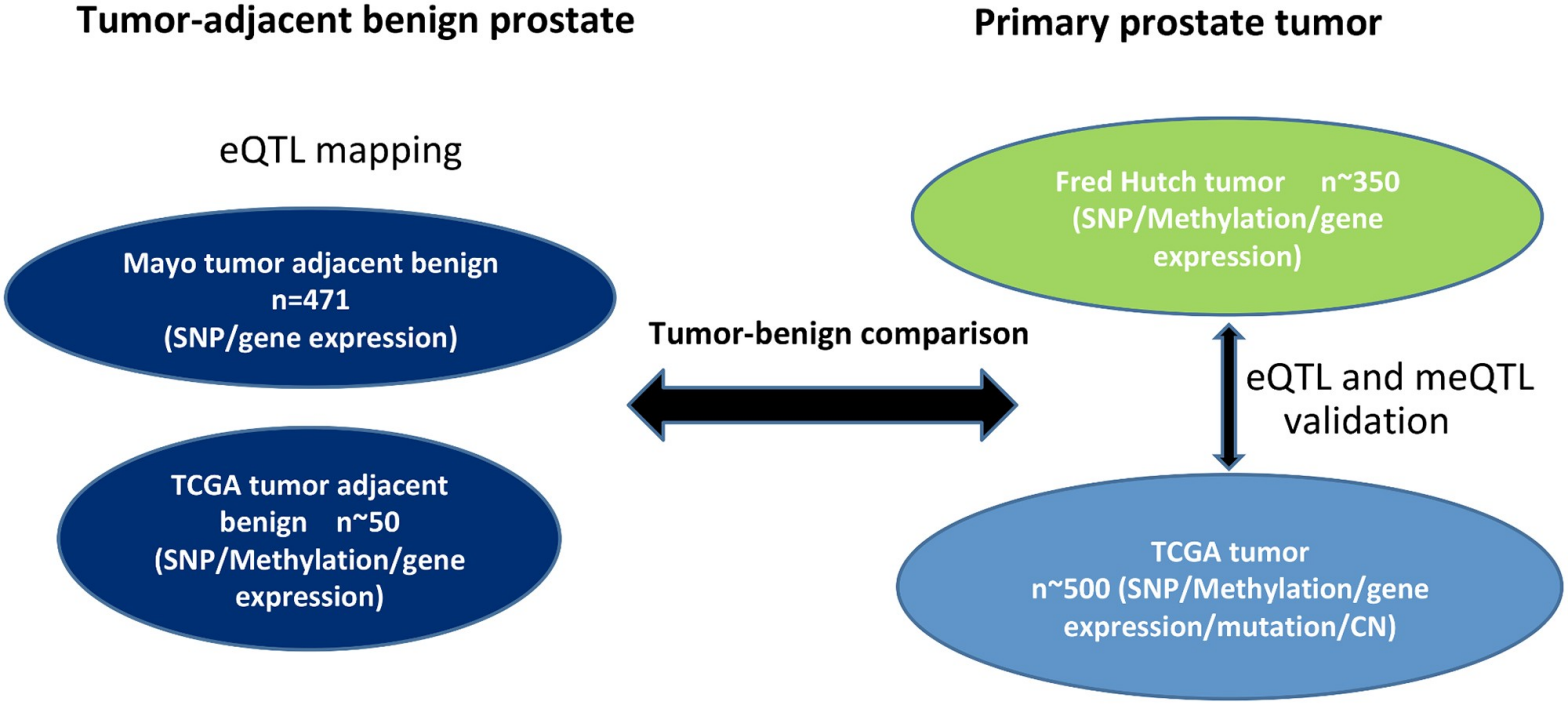
Various samples and genomic data used in this paper.

Fig 2 shows samples, datasets and goals for this analysis. We have included two large sets of primary prostate tumors, both of which have data available on genome-wide genotypes, gene expression and DNA methylation: the first set is from a FH-based cohort of PrCa patients diagnosed with clinically localized stage disease (n = 355), and the second set is the comprehensive genomic data for primary PrCa samples publicly available from TCGA (n~500). Both eQTL mapping and meQTL mapping were conducted in the two tumor datasets. The role of DNA methylation in genetic regulation of gene expression was investigated. For comparison of eQTLs, we have included a previously published set of tumor-adjacent, histologically benign samples from men with PrCa who were treated at the Mayo Clinic (n = 471), which had genome-wide genotypes and gene expression data. The fourth set is the tumor-adjacent, histologically benign samples from TCGA (n = 50), which were compared to the matched TCGA tumor samples, in order to explore whether somatic alterations may explain the differences of eQTL/meQTL mapping results in tumors and in adjacent benign samples. Note that although previous eQTL studies referred to tumor-adjacent, histologically benign samples from cancer patients as “normal” prostate samples [37,38,39], the field effects on somatic alterations (DNA methylation in particular) have been reported [56,57,58]. We therefore referred to the tumor-adjacent samples used in this work hereafter as “benign samples”.

#### *Cis-*eQTL and associated eGenes for 147 PrCa risk SNPs

**Fig 3:**
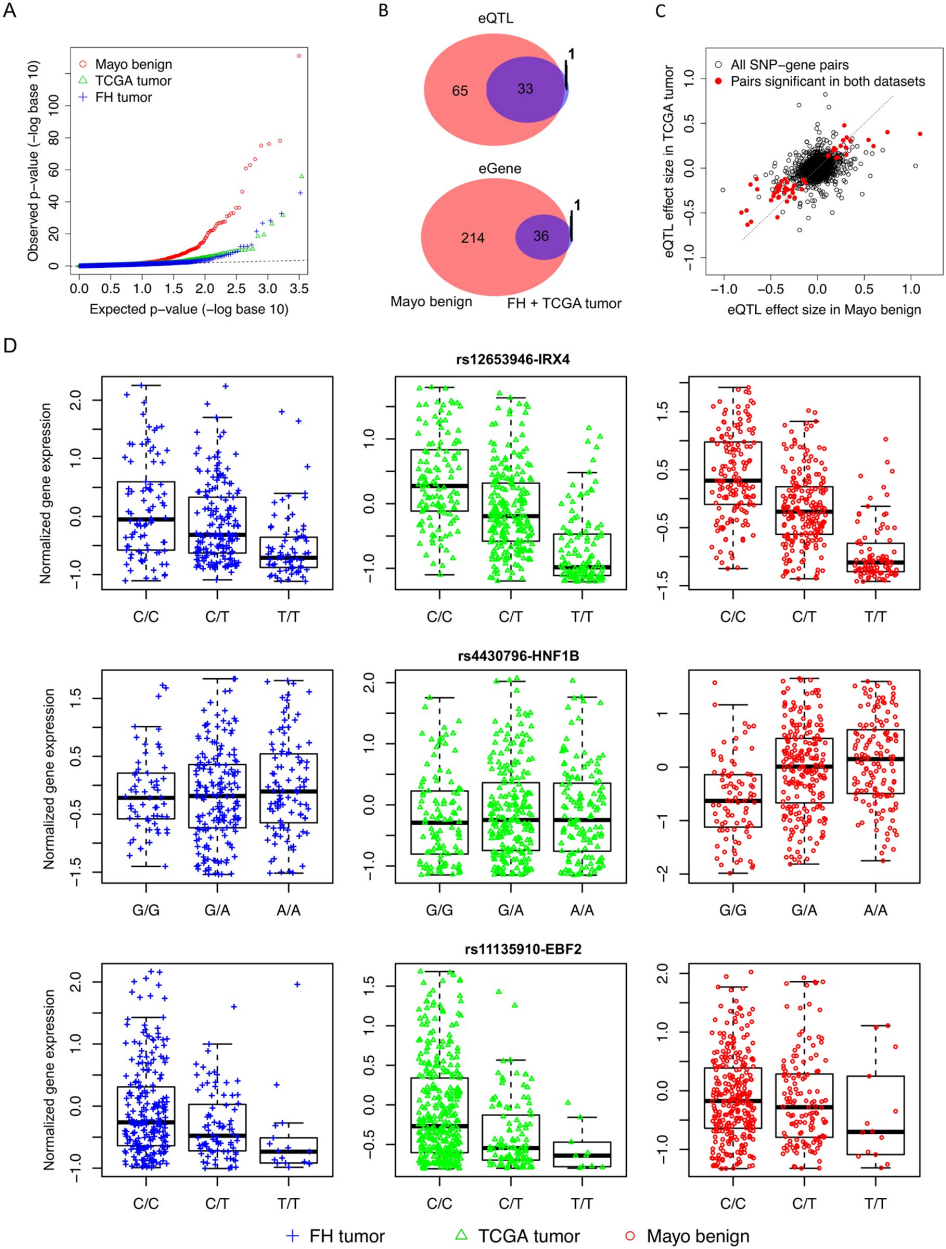
The results of eQTL mapping for the 147 prostate cancer risk SNPs in the Mayo (histologically normal), TCGA (tumor) and FH (tumor) data. **A**, the quantile-quantile plot of SNP-gene association p-values. **B**, the Venn diagram of the eQTLs and eGenes identified in the Mayo histologically normal set and the eQTLs and eGenes confirmed in the TCGA and FH tumor set. **C**, the scatter plot of eQTL effect sizes in Mayo benign samples and TCGA tumor samples. **D**, Standardized gene expressions grouped by genotypes for three examples of eQTL-eGene. The top panel is rs12653946-*IRX4* identified in both tumor and tumor-adjacent histologically normal samples, the middle panel is rs4430796-*HNF1B* only identified in adjacent normal samples, and bottom panel is rs11135910-*EBF2* identified in tumors only.

We first cataloged the total number of possible *cis*-eQTLs for the 147 PrCa risk SNPs, when considering all genes within 1 Mb of each risk SNP. There are 3089 SNP and gene pairs in the Mayo Clinic adjacent benign prostate tissues (n = 471), 3300 pairs in the FH tumor tissues (n = 355), and 3468 pairs in TCGA tumor tissues (n = 492). The different numbers of pairs are due to the different gene expression platforms (gene-expression array for FH samples, and RNA-seq for Mayo and TCGA samples). Fig 3A shows the quantile-quantile plots of p-values resulting from *cis*-eQTL mapping in the three datasets. The eQTL data from the Mayo adjacent benign tissues yielded many more significant p-values than the two tumor datasets, likely due to more eQTLs in histologically benign (non-cancerous) samples. Using the false discovery rate of 0.05 as the significance threshold, Table 1 shows that there are 259 eQTL-eGene pairs (98 eQTL SNPs and 250 eGenes) detected in the Mayo samples, 75 eQTL-eGene pairs (48 eQTL SNPs and 73 eGenes) in the TCGA samples, and 43 eQTL-eGene pairs (34 eQTL SNPs and 42 eGenes) in the FH samples. The details of the significant results for eQTL mapping are shown in S2 Table. The identified eQTLs typically explain a small proportion of gene expression variability: the median R-square for eQTLs in the three datasets is 0.069 (FH), 0.043 (TCGA), and 0.036 (Mayo), respectively.

**Table 1:** Summary of *cis*-eQTL mapping results for 147 prostate cancer risk SNPs in three datasets.

	Mayo adjacent benign (n = 471)	FH tumors (n = 355)	TCGA tumors (n = 492)
#SNP-Gene pairs identified within 1 Mb distance	3089	3300	3468
#eSNP-eGene pairs between FDR <0.05	259	43	75
#eQTL	98	34	48
#eGenes	250	42	73
# eGenes per eQTL (median, min, max)	2,1,12	1,1,3	1,1,7
Distance of eQTL and TSS of the paired eGene (median, min, max)	124764,57,938281	84214,57,808208	60187,57,811509
R-square of eQTL and paired eGene (median, min, max)	0.036,0.017,0.740	0.069,0.037,0.486	0.043,0.024,0.435

In the Mayo adjacent benign samples, 60% of cis-eQTLs were associated with at least two eGenes (median of the number of eGenes associated with an eQTL = 2, max = 12). On average, fewer eGenes were associated with an eQTL in tumor samples (TCGA: median = 1, max = 7, 17 eQTLs associated with more than 1 eGene; FH: median = 1, max = 3, 7 eQTLs associated with more than 1 eGene), suggesting that eQTL regulation may be disrupted in tumors. The top master regulators of gene expression are rs3129859 and rs3096702 from the HLA region in chromosome 6, which are associated with 2 and 9 eGenes in the Mayo set, 7 and 3 eGenes in the TCGA set and 1 and 2 eGenes in the FH set.

A substantial number of eQTLs overlapped between the FH data (19 out of 34) and TCGA data (19 out of 48). The discrepancy between eQTLs found in the two tumor datasets may be explained by differences in sample types used to extract mRNA (FFPE for FH and fresh frozen for TCGA), gene expression profiling methods (microarray for the FH samples and RNA-seq for the TCGA samples) so that some gene expressions are only available in one dataset but not the other, sample sizes and clinical characteristics (TCGA over-sampled high Gleason score tumors and therefore is less representative of primary tumors). Indeed, if one of the two tumor datasets were used as the discovery set (FDR<0.05) and the other dataset as the validation set (p-value <0.05), 25 out of the 34 eQTLs (74%) identified in the FH data were validated by the TCGA data, and 28 out of the 48 eQTLs (58%) identified in the TCGA data were validated in the FH data. Across the two tumor datasets, 39 pairs (34 eQTLs and 37 eGenes) had FDR<0.05 in one dataset and a p-value <0.05 in the other dataset. We therefore define this set of 39 pairs to be the PrCa eQTL-eGene pairs and compare them with the eQTL-eGene pairs in the adjacent benign samples.

We investigated the discrepancy between the FH and TCGA set. For the nine eQTLs and associated 16 eGenes identified in FH but not in TCGA, *ZAK* (paired with rs34925593) and *HCG4P6* (paired with rs12665339) were not available in the TCGA dataset. As the result, two eQTLs- rs34925593 and rs12665339-couldn’t be detected in the TCGA dataset. The rest of 7 eQTLs were associated with 13 eGenes, which were available and expressed in both tumor sets. For the 20 eQTLs and associated 42 eGenes identified in TCGA but not FH, *ZFP57* (paired with rs7767188), *TRIM26* (paired with rs7767188), *MRM2* (paired with rs527510716), *CLDN25* (paired with rs11214775), and *ZNF652* (paired with rs11650494) were not available in the FH dataset. As the result, the four eQTLs (rs7767188, rs527510716, rs11214775 and rs11650494) were not detectable in the FH dataset. The rest 16 eQTLs were paired with 37 eGenes which were available and expressed in the FH dataset. These results suggest most of the discrepancy between eQTLs in FH and eQTLs in TCGA is likely due to differences of sample size and cancer clinical characteristics.

Fig 3B shows the Venn diagrams for the eQTLs and e-Genes identified in the Mayo adjacent benign samples and the tumor samples. One striking finding is that most of the eQTLs identified in prostate tumors were also found in the adjacent benign samples (33 in 34 eQTLs detected in tumors), the majority of eGenes in tumors were also identified in the histologically benign samples, while 34% of 98 eQTLs in the Mayo adjacent benign samples were also detected in tumor samples, suggesting that there were very few tumor-specific eQTL-eGenes. When eQTLs in FH and eQTLs in TCGA were separately compared to eQTLs in Mayo adjacent benign samples, there are 28 eQTLs shared between FH and Mayo, 38 eQTLs shared between TCGA and Mayo. The tumor-specific eQTL-eGene pairs were plotted in S1 Fig. The majority of the tumor-specific eQTLs are likely due to random noise when genotypes only explain a small portion of the variability in gene expressions, so that some SNPs not associated with gene expressions in the benign samples showed a somewhat moderate level of, sometimes inconsistent, association in the two tumor sets (S1 Fig). The difference between the tumor and adjacent normal set is not attributed to the difference of gene-expression platforms. Among 65 eQTL identified in adjacent normal but not in tumor samples, two eQTLs (rs1465618 and rs10086908) have eGenes (LOC100129726 and PCAT1) not available in both the tumor datasets, and 4 eQTLs (rs4713266, rs527510716, rs1512268, rs8008270) have associated eGenes (SMIM13, MRM2, NKX3-1, GNPNAT1) not available in the FH dataset only.

Fig 3C shows the general concordance between effect sizes of SNP-gene associations in TCGA tumors and effect sizes of SNP-gene associations in Mayo benign samples, for all SNP-gene pairs and for the significant eQTL-eGene pairs in both sets. This result suggests that the majority of eQTL regulatory mechanisms in the benign samples are largely intact in tumors, though tumor eQTLs may have smaller effect sizes due to other somatic alterations such as mutations, copy numbers and DNA methylation. Fig 3D shows three representative examples of the most significant eQTL-eGenes, confirming previously reported eQTL mapping results. One pair (rs12653946-*IRX4*) was identified in both tumor and tumor-adjacent benign samples, one (rs4430796-*HNF1B*) was only identified in adjacent benign samples, and the last in tumors only (rs11135910-*EBF2*). Interestingly, all three eGenes encode transcription factors and are believed to be tumor suppressor genes [59–63]. The association of rs1265394 and *IRX4* transcript has been reported previously in Japanese and European populations [59,64]. *HNF1B* is a member of the homeodomain-containing superfamily of transcription factors that may suppress epithelial-to-mesenchymal transition (EMT) in unmethylated, healthy tissues. This tumor-suppressor activity is lost when *HNF1B* is silenced by promoter methylation in the progression to PrCa [61]. It may also be involved in PrCa development via modulating androgenic hormone effects [62]. Consistent with our result, a previous eQTL analysis also reported the eQTL association for rs4430796-*HNF1B* only in tumor-adjacent, histologically benign prostate tissue but not in tumors [38]. The oncogenic role of *EBF2* in PrCa development is less understood [63], though this eQTL association of rs11135910 with *EBF2* has been reported earlier in a smaller subset of TCGA data [65].

Using IPA, a gene set enrichment analysis for the 250 eGenes identified in the Mayo adjacent benign samples was conducted. As the background in these genomic regions, the canonical pathways, molecular functions, and networks enriched by these eGenes were compared to those derived from 2417 genes in the *cis* regions which were not identified to be eGenes of the 147 PrCa risk SNPs. The top ten canonical pathways are all immune—related functions, such as antigen presentation (p-value = 2.2e-10), PD-1, PD-L1 cancer immunotherapy pathway (p-value = 2.0e-7), and allograft rejection signaling(p-value = 3.2e-7), and OX40 signaling(p-value = 4.7e-7, S3 Table). This included numerous *HLA* genes, including *HLA-A*, *HLA-DPB1*, *HLA-DQA1*, *HLA-DQA2*, *HLA-DQB1*, *HLA-DQB2*, *HLA-DRB1*, *HLA-DRB5*, *HLA-G*. These HLA genes are associated with five PrCa risk SNPs in chromosome 6: rs7767188, rs12665339, rs3096702, rs3129859, and rs9296068. The top enriched molecular and cellular functions for these 250 eGenes are important for cell to cell signaling and interaction (24 genes),cell cycle (24 genes), cell morphology (21 genes), cellular development, cellular growth and proliferation (19 genes), and immunological disease (17 genes). The largest molecular functional group are genes related to DNA transcription, including the ones shown in Fig 3d and 3a number of well-known transcription regulators and DNA methylation machineries, such as *ASCL2*, *FOXP4*, *TET2*, *DNMT3B*, *HNF1B*, *HOXA13*, *NOTCH4*, *IRX4*, *CTBP2*, and *ZNF217*. Notably, *TET2* (corresponding eQTL rs7679673) encodes a methylcytosine dioxygenase that catalyzes the conversion of methylcytosine to 5-hydroxymethylcytosine and plays an important role in DNA demethylation. It has been reported that *TET2* binds the androgen receptor and its loss is associated with prostate cancer [66]. *DNMT3B* (corresponding eQTL rs11480453) encodes a DNA methyltransferase which is thought to function in *de novo* methylation and have been implicated in prostate cancer development [67]. Genes involved in steroid synthesis included *CYP21A2*, *HSD17B2*, *ITGA6*, *IDI2*, *MAP2K1*, *PMVK*, *TSPO*, and may correspond to androgen dependent growth of prostate cancer.

#### *cis-*meQTLs and associated CpGs for 147 PCa risk SNPs

**Fig 4:**
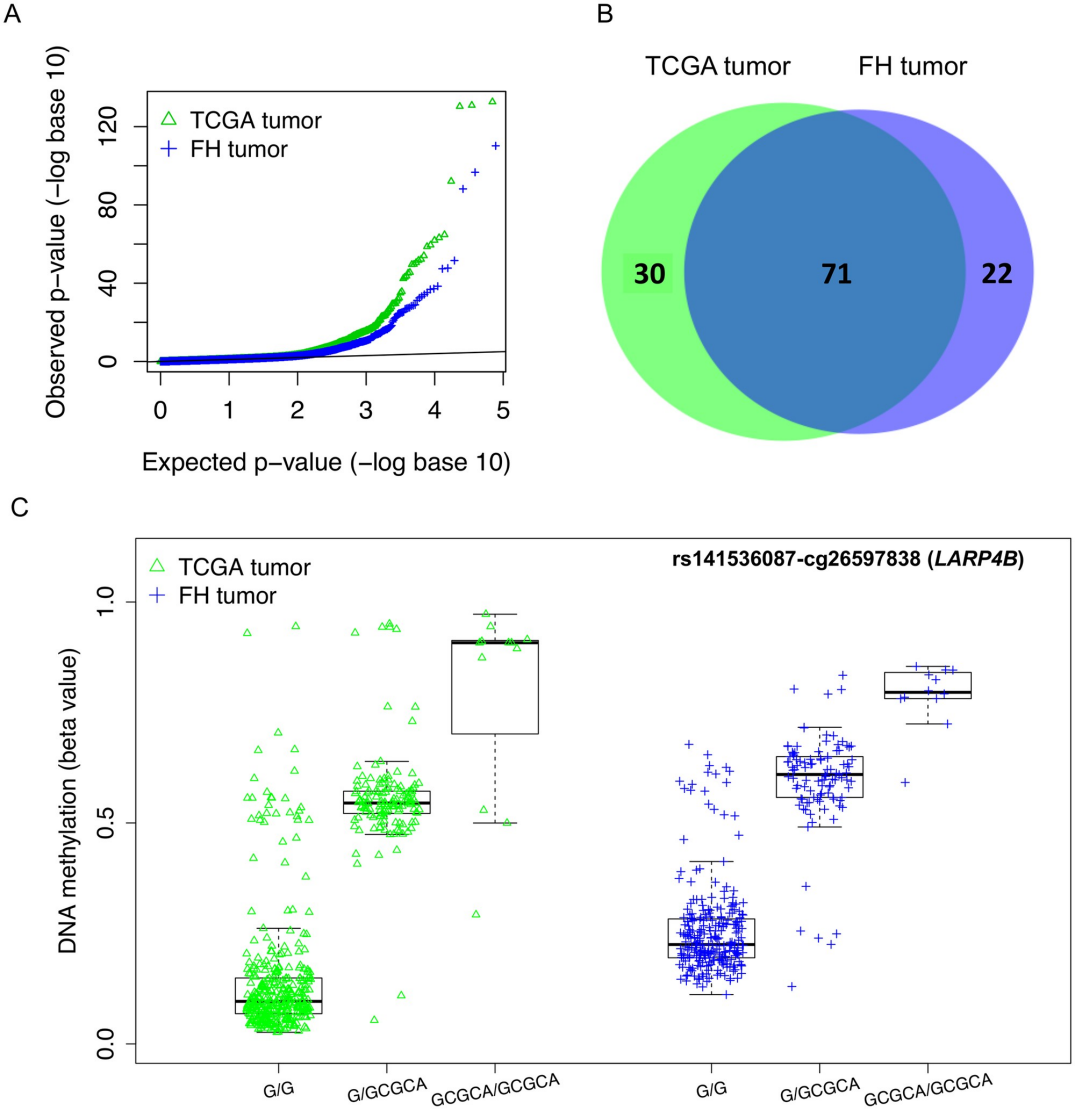
The results of meQTL mapping for the 147 prostate cancer risk SNPs in the FH (tumors) and TCGA (tumors). **A**, the quantile-quantile plot of SNP-CpG association p-values. **B**, the Venn diagram of the meQTL identified in the FH tumor set and the meQTL confirmed in the TCGA tumor set. **C**, beta values of CpG methylation grouped by genotypes for an example meQTL-CpG association.

Within 1 Mb distance of the 147 PCa risk SNPs, 77,649 SNP-CpG pairs were identified in the FH data and 69,602 SNP-CpG pairs in the TCGA data. Cross-reactive and polymorphic CpGs were removed in both datasets. The different numbers of SNP-CpG pairs between the two datasets are due to removal of CpGs in the TCGA dataset that are located within 15bp of a repetitive element. Fig 4 shows the summary of *cis*-meQTL mapping results. The details of the significant results for *cis-*meQTL mapping are shown in S2 Table. The q-q plot in Fig 4A highlights the large number of significant SNP-CpG pairs for both datasets. Table 2 summarizes the *cis*-meQTL mapping results. When FDR <0.05 was used as the significance threshold, approximately two-thirds of the 147 PCa risk SNPs were identified as meQTLs in at least one of the two tumor datasets. There are 776 meQTL-CpG pairs (101 meQTLs and 740 CpGs) identified in the TCGA data, and 586 meQTL-CpG pairs (93 meQTLs and 567 CpGs) identified in the FH data. The median distance between the chromosomal positions of meQTLs and their associated CpGs is 52,747 bp (min = 9, max = 956,913) in the FH dataset, and 67,139 bp (min = 11, max = 985,743) in the TCGA dataset. Similar to eQTLs, meQTLs typically explain a small proportion of the variability of CpG methylation levels: the median R-square for meQTLs in the FH dataset is 0.056, and the median R-square for the meQTLs in the TCGA data is 0.046. The functional annotation of the CpGs linked to meQTLs shows that in the FH data, 13% of the CpGs are located in promoters, 37% in gene bodies (between transcription start site and transcription ending site), and 21% in enhancer regions. In the TCGA data, 16% of the CpGs are located in promoters, 37% in gene bodies, and 17% in enhancer regions. The spatial distribution of these CpGs linked to meQTLs did not differ significantly from all CpGs included on the HM450 array.

**Table 2:** Summary of *cis*-meQTL mapping results for 147 prostate cancer risk SNPs in two datasets.

	FH tumors (n = 355)	TCGA tumors (n = 494)
#SNP-CpG pairs identified within 1 Mb distance	77649	69602
#SNP-CpG pairs with FDR <0.05	586	776
#meQTL with FDR <0.05 (#genes with associated CpGs)	93	101
#CpGs with FDR <0.05	567	740
#CpGs associated with meQTL (median, min, max)	2,1,91	3,1,110
Distance (bp) between meQTL and associated CpGs (median, min, max)	52747,9,956913	67139,11,985743
R-square of meQTL and associated CpGs (median, min, max)	0.056,0.035,0.757	0.046,0.025,0.738

Typically, meQTLs are associated with multiple CpGs (median = 2, min = 1, max = 91 in the FH data, and median = 3, min = 1, max = 110 in the TCGA data). These meQTLs may be master regulators of DNA methylation that involves multiple genes. Among 93 meQTLs identified in FH, 26 of them are associated with multiple CpGs which are located in more than one gene. Among 101 meQTLs identified in TCGA, 33 of them are associated with multiple CpGs which are located in more than one gene. Notably, we found the three PrCa SNPs in chromosome 6 (rs7767188, rs3129859 and rs3096702) are master regulators of DNA methylation in over 9 genes in near regions. All three SNPs are also identified as eQTLs in TCGA and FH tumor data.

Fig 4B shows that meQTLs (with FDR <0.05) identified in the two datasets are highly concordant, with 71 meQTLs (76% of FH meQTLs) being shared. The concordance increases to 85 meQTLs (91% of FH meQTLs) if we use a less stringent significance rule: FDR <0.05 in one dataset and p-value <0.05 in the other. This level of consistency is much higher than the eQTL findings in the two datasets, which may be attributed to the better preservation of DNA than RNA in FFPE tumor tissue samples, and the fact that the same profiling method was used for measuring DNA methylation (Illumina HM450 BeadChip).

Fig 4C shows an example of meQTL-CpGs appearing in both the FH and TCGA sets. SNP rs141536087 is located in the gene-body of *LARP4B*, and cg26597838 is located in an enhancer region approximately 17 Kb upstream of *LARP4B*. *LARP4B* is an RNA binding protein that has been previously identified as a putative tumor suppressor that inhibits cell migration and invasion of prostate cancer cells [68]. Furthermore, rs141536087 is an eQTL for *LARP4B* in the Mayo set (p-value = 4.5e-15) but not in the two cancer sets (p-value = 0.41 for FH and 0.63 for TCGA).

#### Relationship between *cis-*eQTL and *cis*-meQTL

**Fig 5:**
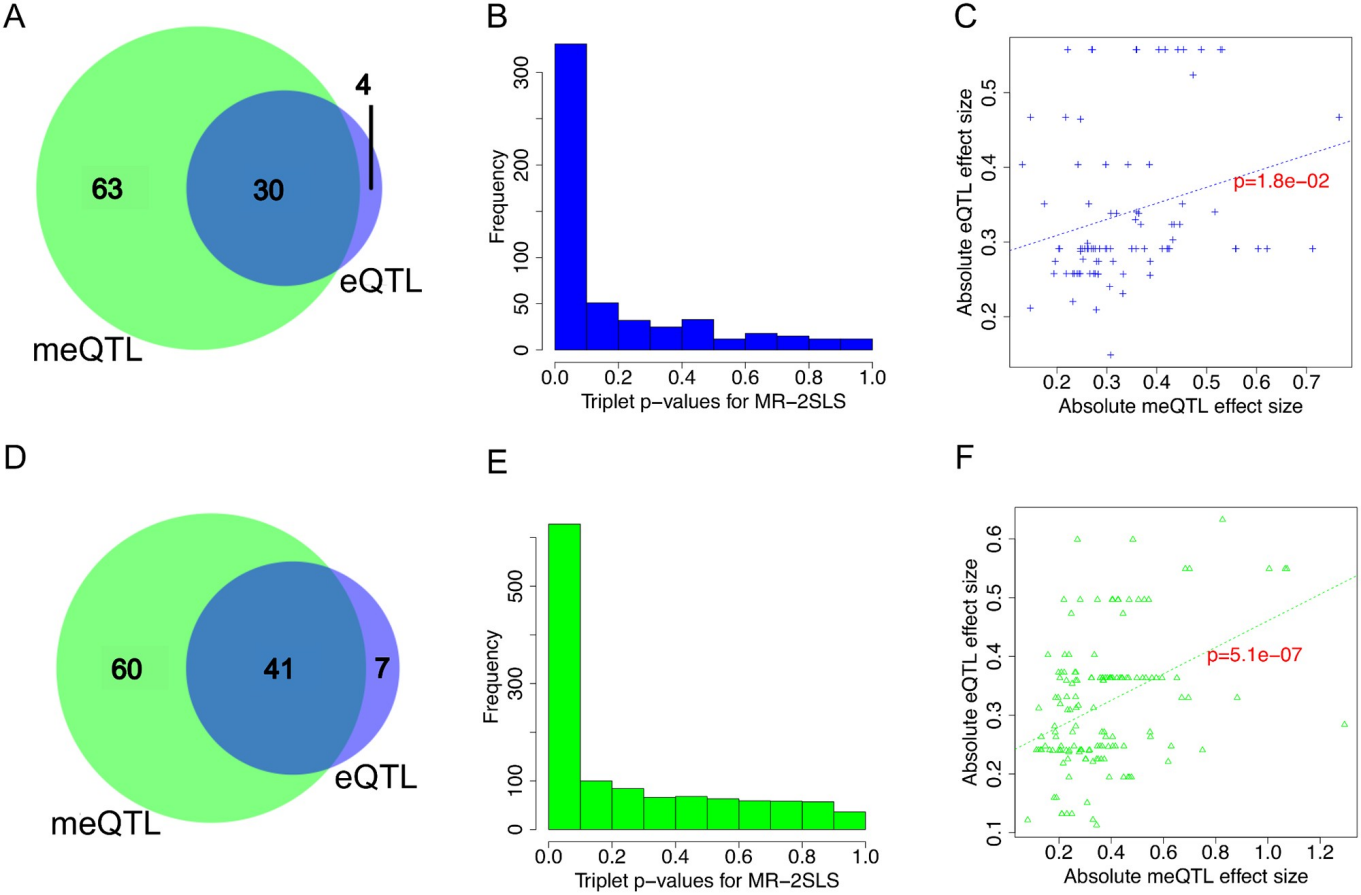
Genetic regulation of gene expression overlaps with genetic regulation of CpG methylation in the 147 PrCa risk SNPs. **A**, the Venn diagram of eQTL and meQTL identified in the FH tumor set. **B**, the histogram of p-values when applying the two-stage least squares method to FH SNP-CpG-gene expression triplets when SNPs are both eQTL and meQTL. **C**, the scatter plot of genetic associations with gene expression and genetic associations with CpG methylation for SNPs identified as eQTL and meQTL in the FH tumor set and CpGs located in the same gene of gene expression. **D**, the Venn diagram of eQTL and meQTL identified in the TCGA tumor set. **E**, the histogram of p-values when applying the two-stage least squares method to FH SNP-CpG-gene expression triplets when SNPs are both eQTL and meQTL. **F**, the scatter plot of genetic associations with gene expression and genetic associations with CpG methylation for SNPs identified as eQTL and meQTL in the TCGA tumor set and CpGs located in the same gene of gene expression.

Fig 5 includes the Venn diagram of eQTLs and meQTLs identified in the two tumor datasets. The majority of eQTLs are also meQTLs in tumor datasets (Fig 5A and 5D), more so in the TCGA data (30 out of 34 in the FH data; 41 out of 48 in the TCGA data), while only ~41% of meQTLs are also eQTLs in the TCGA data, and ~32% of meQTLs are also eQTLs in the FH data. One complicating factor for comparing eQTLs and meQTLs is that there were more pairs being tested for meQTL than eQTL pairs, therefore meQTL mapping is penalized by a greater degree of multiple testing correction. If a less stringent significance threshold (FDR <0.2) is used for meQTL mapping, 44 out of 48 eQTLs identified in the TCGA data are also meQTLs, and 32 out of 34 eQTLs in the FH data are also identified as meQTLs, reinforcing the finding that eQTLs is mostly a subset of meQTL in tumor sets.

For those SNPs identified as both eQTL and meQTL, causal effects of the associated CpG methylation sites on the corresponding gene expressions were assessed for 541 SNP-CpG-gene expression triplets (31 SNPs, 385 CpGs, 39 genes) in the FH set and 1219 SNP-CpG-gene expression triplets (41 SNPs, 539 CpGs, 72 genes) in the TCGA set, using the Mendelian randomization (MR) method exploiting genetic variants as instrumental variables. Fig 5B and 5E show the histograms of p-values derived from the two-stage least squares method for the two tumor sets, both showing a high proportion of triplets with evidence of causal effect (47% triplets in FH have FDR<0.05, 35% triplets in TCGA have FDR<0.05). However, a significant MR result could also due to the genotype affecting DNA methylation and gene expression independently. An alternative method is to assess the dose correspondence of eQTL effect sizes and meQTL effect sizes. The triplets were restricted to those containing CpG sites located in the same genes whose expression was measured (91 in FH and 145 in TCGA), Fig 5C and 5F assess the concordance of the absolute values of genetic associations with standardized CpG methylation levels and the absolute values of genetic associations with standardized gene expression levels. The absolute values of coefficients were plotted because DNA methylation can be positively or negatively associated with gene expression, depending on the locations of the CpGs in a genic region (gene body or promoter). Both scatter plots show the dose response relationship between a SNP’s genetic influence on DNA methylation and on gene expression (p-value for a linear trend is 0.018 for the FH data and 5.1e-7 for the TCGA tumor data), which provides evidence of a causal relationship between DNA methylation and gene expression. Taken collectively, the results in Fig 5C and 5F suggest that genetic regulation of gene expression and genetic regulation of CpG methylation are not independent molecular events, though neither Mendelian randomization nor the dose response can differentiate the direction of the causal effect: it could be that eQTL associations were mediated by altering DNA methylation in the respective genes, or that DNA methylation is a downstream event after a genetic variant affecting gene expression.

#### Absence of eQTLs in tumor samples may be due to altered DNA methylation

The TCGA genomic data for 50 pairs of primary PrCa and adjacent benign samples were investigated for reasons that may explain the “loss” of eQTLs when comparing tumors to adjacent benign samples. We investigate whether differential gene expression, copy number alterations or somatic mutations between tumor and normal samples where associated with loss of eQTL in tumors. There is no systematic difference for either of three genomic features when we compare 53 genes which lost eQTL control in TCGA tumors to 173 eGenes whose eQTL regulation remain intact in TCGA tumors.

**Fig 6:**
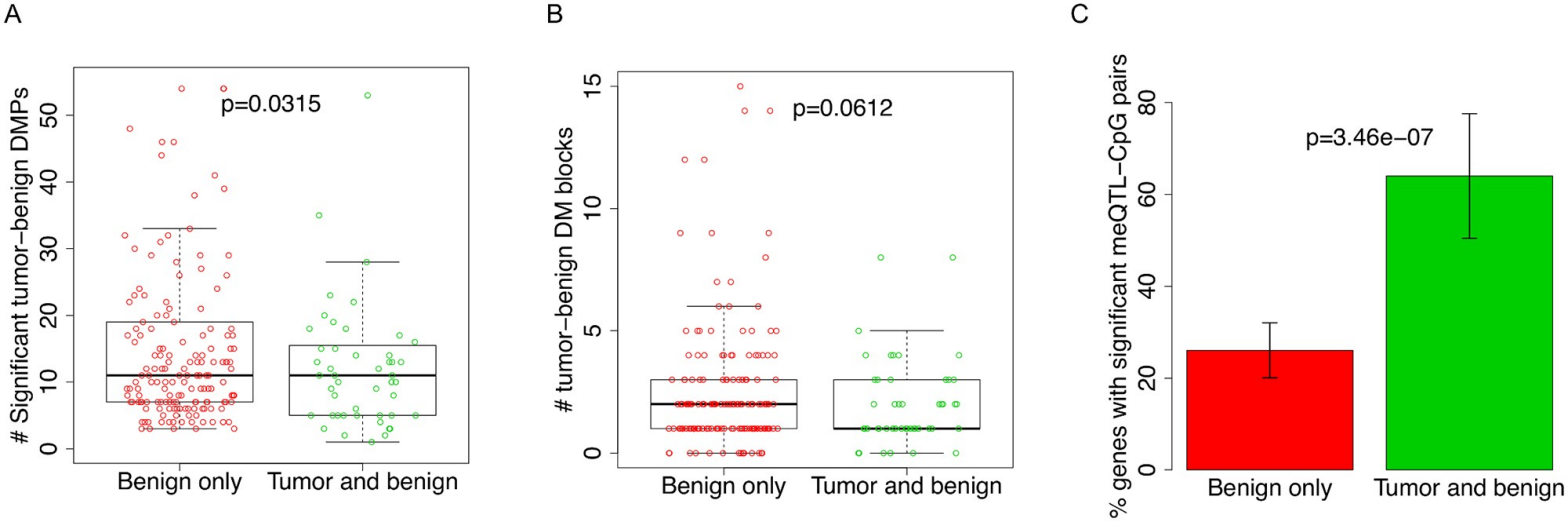
Altered DNA methylation in genes which lose genetic control in tumors when compared to benign samples. **A**, Number of differentially methylated probes (DMP) in the eGenes in benign samples only and eGenes in both tumor and benign samples. **B**, Number of differentially methylated blocks in eGenes in benign samples only and eGenes in both tumor and benign samples. **C**, the percentage of genes associated with at least one meQTL-CpG pair within the genes.

Fig 6 shows the comparison of DNA methylation in the eGenes in adjacent benign samples only and the eGenes in both tumor and benign samples. DNA methylation data available for the paired 50 tumors and 50 tumor-adjacent benign samples were investigated in two ways. First, differentially methylated probes (DMP) between tumor samples and tumor-adjacent, histologically benign samples (paired with matched tumor) were identified in regions surrounding eGenes. There are more differentially methylated probes between tumors and adjacent benign samples around the eGenes that “lost” genetic regulation in tumors from eQTLs compared to eGenes that “maintained” genetic associations in both benign samples and tumor samples (Fig 6A, p-value = 0.032). Second, the differentially methylated region (DMR) between tumor samples and tumor-adjacent, histologically benign samples were determined by at least two consecutive probes with significant differences in the same direction. Fig 6B compares the number of DMRs in two groups of genes: there are more tumor-benign DMRs in the genes which lost genetic regulation in TCGA tumors, with a marginally statistically significant difference (p-value = 0.0612). Finally, the percentage of genes containing at least one meQTL-regulated CpG sites (as defined in the TCGA set in Fig 4) was compared between the two groups of eGenes. Consistently, there was a substantially higher percentage of eGenes in tumor and benign samples which have genetically regulated CpGs, when compared to the eGenes in benign samples only (Fig 6C, 64% vs 26%, p-value = 3.46e-7). In S2 Fig, two examples were shown where eQTL associations were evidently weakened in the tumor data and, simultaneously there are substantial differences between DNA methylation at key CpG sites within the gene between tumors and adjacent benign samples (~50 pairs from TCGA). Together with Fig 5, these results in Fig 6 and S2 Fig suggest the hypothesis that at least some altered CpG methylation sites may be mechanistically involved in loss of genetic regulation of gene expression in tumor sets.

#### Mediation analysis of genetic influence on DNA methylation and gene expression

**Fig 7:**
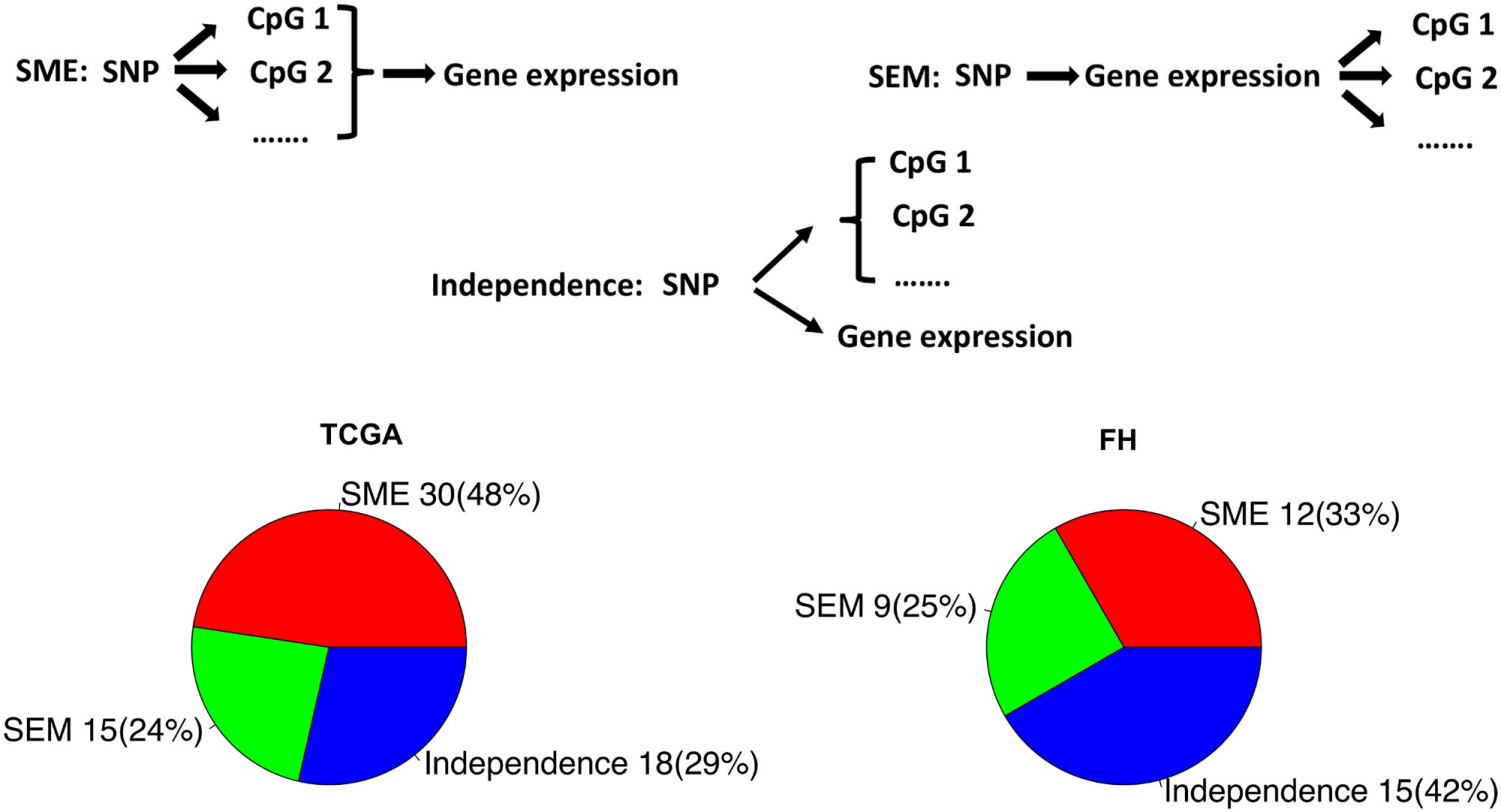
Three possible SNP-Methylation-gene expression relationships and representative results of mediation analyses from TCGA and FH.

To further dissect the direction of causality among the three possible relationships (Fig 7), mediation analyses between PrCa risk SNPs, CpG methylation probes and expressions of eGenes were conducted using the Causal Inference Test (*CIT*) method [55] and the three additional criteria we propose. *CIT* is a four-component composite test that was designed to test mediation of the effect of an exposure on an outcome by an intermediate phenotype (see details in the Methods Section). The goal here is to identify three types of mediation relationships: 1) a SNP influences gene expression and DNA methylation through independent pathways (denoted as independence, no causal relationship); 2) a SNP alters CpG methylation, which in turn influences gene expression (SNP→Methylation→Gene, denoted as the SME mediation); and 3) a SNP changes gene expression, which unwind local chromosome and triggers passive alterations in CpG methylation (SNP→Gene→Methylation, denoted as the SEM mediation). See the top panel in Fig 7 for illustration of the three tri-variate relationships. The unique feature is that there will be typically multiple CpG sites involved in any eQTL-eGene pair.

In the TCGA dataset, 41 risk SNPs identified as both eQTL and meQTL were included in the mediation analysis, together with their associated CpG probes and gene expression (72 eGenes, 539 CpG probes). There are 1219 QTL-CpG-Gene triplets, 824 of them have both *CIT*-SEM and *CIT*-SME p-values > 0.05, 210 have both *CIT* p-values <0.05, 185 have one of the two *CIT* p-values <0.05. Pooling multiple CpGs in the *cis*-region around a gene together and using CIT and three proposed criteria to differentiate SME and SEM, we identified evidence of SME for 30 QTL-CpGs-Gene relationships, 15 SEM triplets for QTL-Gene-CpGs relationships, and 18 independent QTL-Gene/QTL-CpGs relationships (Fig 7). In the FH dataset, 31 risk SNPs identified as both eQTL and meQTL were included in the mediation analysis, together with their associated CpG probes and gene expression (39 eGenes and 385 CpG probes). There are 541 QTL-CpG-Gene triplets, 394 of them have both *CIT*-SEM and *CIT*-SME p-values > 0.05, 99 have both *CIT* p-values <0.05, 48 have one of the two *CIT* p-values <0.05. Pooling nearby CpGs, we identified 12 SME triplets, 9 SEM triplets, and 15 independent triplets in the FH set (Fig 7). The two pie plots in Fig 7 shows the distribution of the three relationships. Across the two datasets, over two-thirds of the triplets showed evidence of a causal relationship, the majority of which are SME. The details of mediation results are shown in S4 Table.

Table 3 shows examples of the SNP-CpGs-eGene triplets that were identified to be SME (5 triplets) or SEM (4 triplets), and that were consistent between FH and TCGA datasets. This group of risk SNPs provides the strongest evidence of that regulation of the corresponding eGenes involves alterations of DNA methylation. The majority of these SNPs in Table 3 have been previously reported to be eQTLs for some putative PrCa risk genes, including rs2292884 for *MLPH* [69], rs12653946 for *IRX4* [59,60], rs1933488 for *RGS17* [70,71], rs10993994 for *MSMB* [72,73], rs5945619 for *NUDT11* [38,74], Notably, SNP rs684232 has been reported to be associated with *FAM57A* gene expression [38,39], a gene encoding membrane-associated protein that promotes lung carcinogenesis [75], though its role in PrCa has not been reported. Several pairs of eQTL-eGenes represent new discoveries. SNP rs10875943 is between the tubulin gene *TUBA1C* and the peripherin gene *PRPH*. Our result suggests its link to *TUBA1C*, though its functional role in PrCa has not been studied. The genes in the 6p21/MHC region, *HLA-DQB1* and *HLA-DRB5*, that are associated with rs3096702 and rs3129859, have not been previously studied in relation to PrCa.

**Table 3:** Examples of the triplets (SNP, CpGs, eGene) with a mediation relationship (either SME or SEM) that is consistent in FH and TCGA datasets.

SNP	Chr (position)	Associated CpGs, overlapped between FH and TCGA	CpGs regulatory region	eGene (TSS position)	Mediation type	Median *CIT* p-value (FH;TCGA)	Proportion explained by CpGs if SME(FH;TCGA)
rs2292884	2 (238443226)	cg00285317, cg27051686	Enhancer/DHS	*MLPH* (238395053)	SME	0.009;0.002	23%; 81%
rs3096702	6 (32192331)	cg07180897,cg15343510	Gene body	*HLA-DQB1* (32627240)	SME	0.005;0.003	77%;81%
rs3129859	6 (32400939)	cg12672189, cg05383619	TSS1500, gene body, DHS	*HLA-DRB5* (32485153)	SME	0.01;0.007	100%; 68%
rs12653946	5 (1895829)	cg00089823, cg00483562,cg00626856, cg03587843,cg04849541,cg06161964, cg09672187,cg11279838,cg13143349, cg14051264,cg14823763,cg16210248,cg17650747, cg18764814,cg26195178	DHS/DMR	*IRX4* (1877540)	SME	0.002;0.003	54%; 100%
rs1933488	6 (153441079)	cg03661775,cg16924337,cg17264670,cg19904233, cg22867315,cg23651356,cg24028809,cg24312610	Enhancer/DHS	*RGS17* (153332031)	SEM	0.001;1e-4	-; -
rs10993994	10 (51549496)	cg00807366	Enhancer	*MSMB* (51549552)	SEM	0.023;0.002	-;-
rs10875943	12 (49676010)	cg04797936,cg12073537,cg22606869,cg25751371	Promoter-associated	*TUBA1C*	SME	0.002;1e-4	34%;84%
rs684232	17 (618965)	cg13073302,cg25186143	TSS1500, gene-body	*FAM57A*	SEM	0.008;6e-4	-;-
rs5945619	X (51241672)	cg16065628	Gene-body, north shore	*NUDT11*	SEM	0.002;0.003	-;-

**Fig 8:**
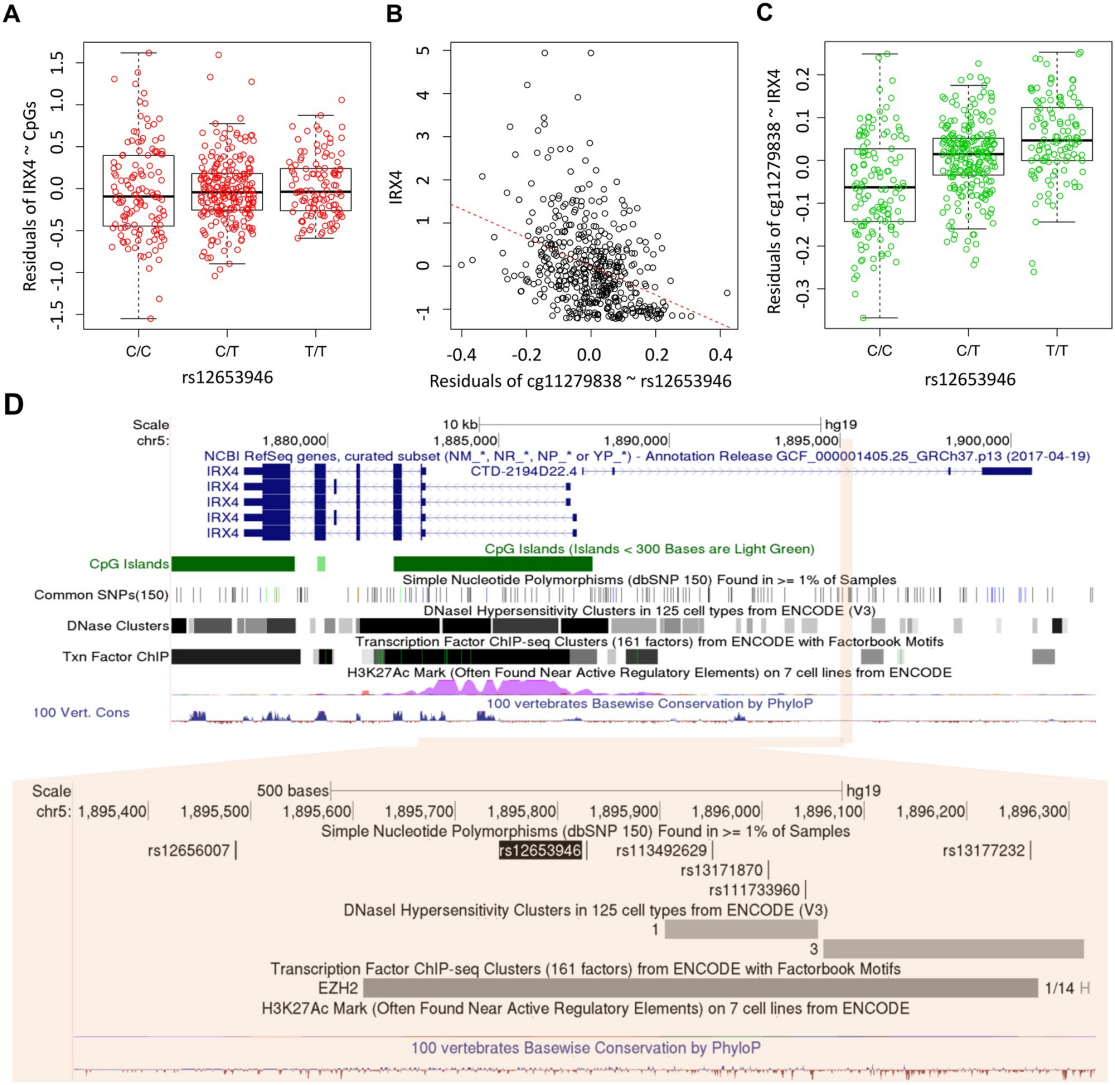
An example of the mediation relationship: rs12653946 and *IRX4* in chromosome 5. **A**, residuals of gene expression~CpG versus genotype. **B**, gene expression versus residuals of CpG~genotype. **C**, residuals of CpG~gene expression versus genotypes. **D**, the genomic annotation maps by UCSD genome browser for the region with rs12653946 and *IRX4* in chromosome 5.

**Fig 9:**
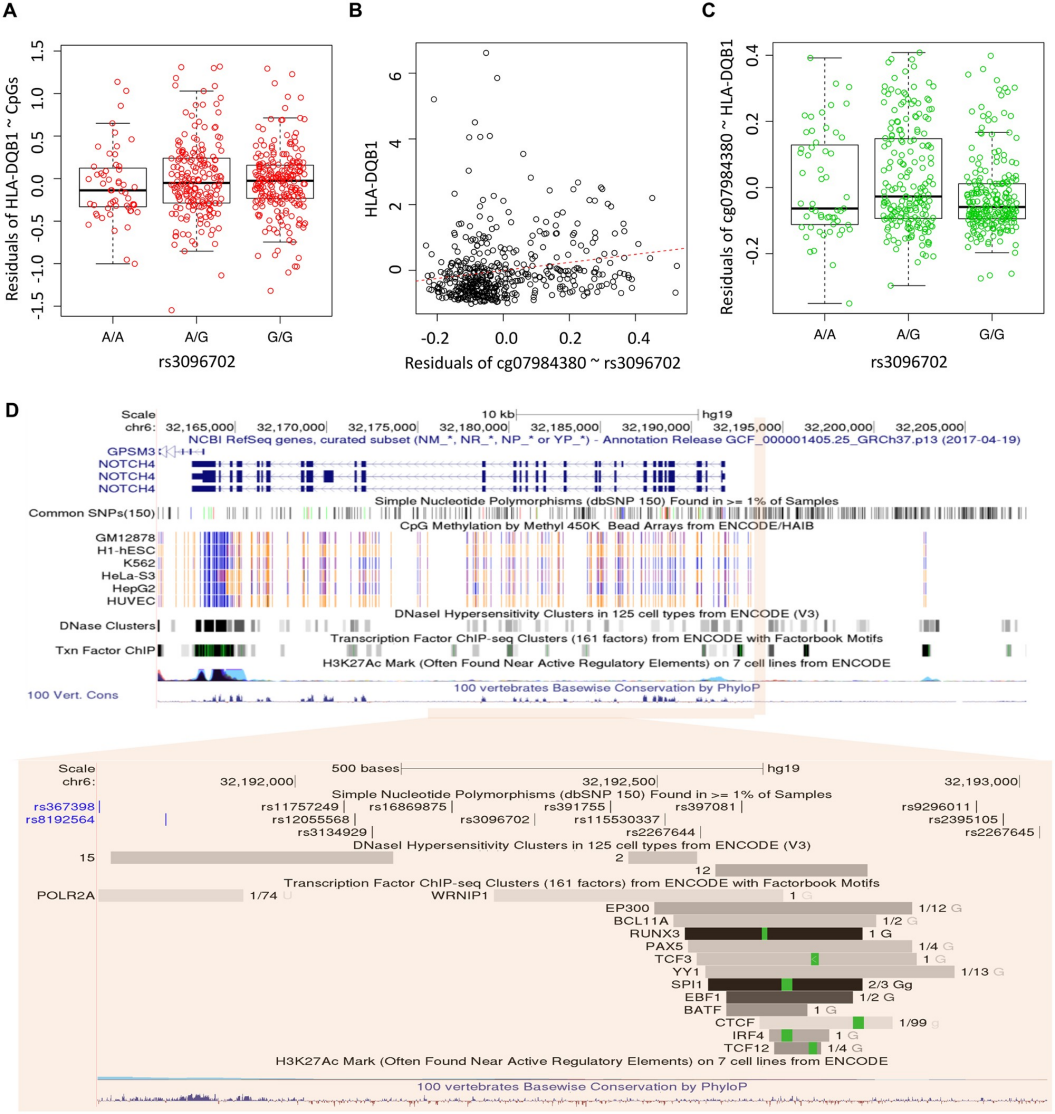
Another example of the mediation relationship: rs3096702 and *NOTCH4* in chromosome 6. **A**, residuals of gene expression~CpG versus genotypes. **B**, gene expression versus residuals of CpG~genotype. **C**, residuals of CpG~gene expression versus genotypes. **D**, the genomic annotation maps by UCSD genome browser for the region with rs3096702 and *NOTCH4* in chromosome 6.

A substantial proportion of the mediation relationships for the triplets are inferred to be SME (Fig 7), evidence of the mechanistic role of DNA methylation in the genetic regulation of gene expression. Figs 8 and 9 shows two examples of SME with three diagnostic plots for inferring causal direction: residuals of gene expression~CpGs regression versus genotypes (Figs 8A and 9A), gene expression versus residuals of CpG~genotype regression (Figs 8B and 9B), and residuals of CpG~gene expression regression versus genotypes (Figs 8C and 9C). Associations shown in the last two plots but not the first plot indicate a SME relationship. Specifically, rs12653946 is located in an intronic region of an RNA gene transcribed upstream of *IRX4*, regulating gene expression of *IRX4* [59,60] (Fig 8). Our result suggests that this gene regulation is mediated altered DNA methylation in the CpG islands located in the first exon and the gene body. For the 6p21/MHC region (Fig 9), previously SNP rs3096702 was suggested to be associated with *NOTCH4*, a nearby gene that may be related to epithelial-mesenchymal transition (EMT) and PrCa growth [76]. Our result suggests that this SNP influences gene expression of an HLA class II gene *DQB-1*, which may be related to immune escape of PrCa. Furthermore, this genetic influence on gene expression is mediated through multiple CpGs in the gene body and TSS1500 region. The proportion of genetic control on gene expression explained by the CpGs was as high as 50% ~ 100% in the two tumor sets for the two genes. Further inspection of ENCODE data in the 30 kb region surrounding this SNP suggests that rs3096702 is in binding sites for multiple transcription factors and the transcriptional regulator protein *CTCF*, 300 kb upstream of the *HLA* gene. These results suggest that rs3096702 may affect transcription binding affinity and enhancer-mediated epigenetic machinery, then regulate the methylation and gene expression of *HLA* genes.

## Discussion

In this work we performed rigorously eQTL and meQTL mapping for the 147 confirmed PrCa risk SNPs using comprehensive genomic data in primary prostate tumors (TCGA and FH) and tumor-adjacent benign samples (Mayo Clinic). To our knowledge this is the first eQTL study for PrCa risk SNPs that also includes DNA methylation data, and the first to systematically investigate the differences of eQTLs in prostate tumors and tumor-adjacent benign samples. Methodologically, we also carefully examined the existing approaches and proposed a modified version of CIT to disentangle the role of DNA methylation in eQTL regulation. The impact of this work is primarily on the prostate cancer literature and the functional interpretation of 147 prostate cancer risk SNPs. The contributions of this work are two folded: we systematically compare eQTLs and meQTLs among prostate cancer tissue and the adjacent normal tissue which has not been done previously; we rigorously dissect the role of DNA methylation in eQTL regulation of gene expression. Several important observations were made as discussed below.

First, perhaps not surprisingly, there are many more eQTLs identified in the tumor-adjacent benign samples than in primary tumor samples. Moreover, nearly all eQTLs in tumors are also identified as eQTLs in tumor-adjacent benign samples, yet approximately half of eQTLs in adjacent benign samples were not present in tumors. This observation is consistent with previous observations that eQTL association signals in normal or benign samples tend to be attenuated in tumor samples [37], if not absent, and benign tissue samples tend to have more eQTLs than tumor samples [77]. One possible explanation for an eQTL only identified in histological benign samples but not in tumors is that the corresponding eGene may only function in tumor initiation, become silenced in tumor progression. For example, our result (Fig 3D) confirmed previous findings that rs4430796 is an eQTL for *HNF1B* only in the tumor-adjacent benign samples [38,77], but not in tumor samples. Recent functional assays suggest that *HNF1B*, which encodes a transcription factor, is a pro-differentiation factor that suppresses epithelial-to-mesenchymal transition (EMT) in unmethylated, healthy tissues [61]. This tumor-suppressor activity is lost when *HNF1B* is silenced by promoter methylation in the progression to PrCa, and it is therefore no longer associated with the eQTL [61]. Along this same line, it is also possible that somatic alterations such as mutations, copy number changes and aberrant DNA methylation arising in the tumor genome perturb gene expression, which may disrupt or weaken eQTL associations. This explanation is consistent with Fig 3C, where there is a global concordance between eQTL effect sizes in benign samples and eQTL effect sizes in tumors. We have thoroughly investigated the TCGA genomic data regarding somatic alterations in the PrCa genome, and we show in Fig 6 that the loss of eQTL regulation in gene expression in tumors may be due to widely altered DNA methylation in tumors, less likely due to somatic mutations or copy-number alterations. On the other hand, we conclude that among the 147 PrCa susceptibility SNPs, tumor-specific eQTLs are very rare, likely because the eQTLs in these susceptibility SNPs predispose to cancer risk through influencing oncogenic genes in benign samples during the early stage of tumor development. One complication factor is the field effect of carcinogenesis–the benign prostate samples are tumor-adjacent, histologically benign, taken from cancer patients. It is possible that gene expression profiles of tumor-adjacent, histologically benign samples have been altered by the tumor field effect.

Second, the majority of the 147 risk SNPs (~100) were identified to be meQTLs in prostate tumors, yet only a subset was identified as eQTLs. Intriguingly, nearly all eQTLs were also identified as meQTLs. It is known that aberrant DNA methylation play a key role in PrCa development, which may interfere with genetic regulation of gene expression. We hypothesize that, if we indeed had a large set of normal prostate samples, we would observe more PrCa SNPs that are meQTLs in normal prostate samples, with a high degree of overlap between eQTLs and meQTLs in normal prostate tissue. This level of concordance is much higher than what was reported in the recent genome-wide analyses of eQTLs and meQTLs from peripheral blood and lymphoblastoid cell lines [48–51], suggesting that genetic regulation of gene expression by these PrCa risk SNPs are very much intertwined with methylation changes, either actively as a mediator or passively as a downstream consequence. Our results in Fig 4 and mediation analyses support this hypothesis. There are several possible explanations for the observation that more risk SNPs are meQTLs than eQTL. It is known that DNA methylation may have broader biological functions in maintaining chromosomal stability and cellular differentiation beyond regulating gene expression. Furthermore, mRNA abundance is much more dynamic, subject to multiple regulating mechanisms and more liable to measurement error when compared to DNA methylation. Therefore, eQTL mapping may be inherently more variable than meQTL mapping. Thus, not all meQTLs become eQTLs in a particular cellular state, similar to the observation that not all differentially methylated CpGs correlates with altered gene expression.

Third, among the risk SNPs that were both eQTLs and meQTLs, our mediation analysis suggests that the majority of triplets (SNP, DNA methylation, gene expression) display a causal mediation relationship, either of SNP→Methylation→Expression (SME) or SNP→Expression→Methylation (SEM), supporting the important role of DNA methylation in PrCa risk SNPs for regulation of gene expression. DNA methylation variable sites are known to be associated with gene expression mechanistically in complex and context-dependent ways, which includes both active (e.g., DNA hypomethylation causally affecting gene expression through transcription factor binding) or passive methods where DNA methylation is a consequence or independent mark of gene expression (e.g., reflecting the chromatin state) [49]. Statistically it is challenging to discern whether DNA methylation status is an active or passive consequence of gene expression. For example, standard *CIT* could not distinguish the SME from the SEM model in a recent genome-wide eQTL and meQTL analysis.^53^ Leveraging typically multiple candidate mediating CpG sites in a gene, we have added additional discriminatory criteria to the *CIT* test in order to separate SME from SEM: 1) the SME p-value is smaller than the SEM p-value, and the proportion of association explained by SME should be greater than SEM; and 2) the proportion of genetic association explained by SME should be greater if multiple CpGs are included in mediation analysis. These criteria are helpful to disentangle the causal direction, though its exploratory nature requires caution in interpretation. Altogether, these data support the important role of altered DNA methylation as a mechanism for the influence of these 147 SNPs on gene expression.

Among the eGenes that are regulated by the 147 PrCa risk SNPs in benign tissue, the immune response pathways stand out as the most significantly enriched. This observation corresponds to the risk SNPs in chromosome 6 and various HLA genes located in the region, reiterating the importance of immune responses in the early developmental stage of PrCa. Prostate cancer cells produce a number of tumor associated antigens (TAA) [78,79,80], such as prostate-specific antigen (PSA), prostate acid phosphatase (PAP), and prostate-specific membrane antigen (PSMA). The typically slow growth of prostate tumors allows time for the immune system to mount an anti-tumor response to these antigens. Our eQTL analysis supports the hypothesis that polymorphisms of the HLA alleles are associated with expression levels of various HLA molecules and, likely, the efficiency of immune response to particular TAAs and the risk of PrCa.

While this work presents a comprehensive analysis of eQTLs and meQTLs among 147 SNPs using multiple large genomic datasets, one weakness of our meQTL analysis is that we did not have a large set of normal prostate tissue samples with genome-wide DNA methylation data. This limits our capability to examine the relationship between SNPs, DNA methylation and gene expression in normal prostate. It remains of interest to determine whether the intermediary role of DNA methylation in regulation of gene expression that we observed in tumor samples is consistent in normal prostate samples. Strictly speaking, the tumor-adjacent, histologically benign samples in the Mayo dataset may already have some cancer-related molecular alterations (these benign samples were obtained from patients with PrCa), therefore are not ideal for a tumor-normal comparison study. However, it is difficult to obtain an adequate amount of normal prostate tissue samples through biopsy or donors, e.g., GTEx has a limited number of normal prostate samples. Finally, a limitation for our eQTL analysis is that the FH data were generated using FFPE tissue samples and array-based methods, which may not be ideal for measuring low abundance of mRNA and gene expression. In addition to the difference of clinical characteristics between TCGA and FH data (TCGA has many more high-grade tumors), this factor may also contribute to the differences in eQTL results between FH and TCGA, since the latter used RNA obtained from fresh frozen samples.

In conclusion, we conducted comprehensive eQTL and meQTL analyses for 147 PrCa risk SNPs, in tumors and tumor-adjacent benign samples, and we investigated the role of DNA methylation in eQTL regulation of gene expression. The eQTLs and associated eGenes provide insight into the molecular biology of PrCa, and there is strong evidence that DNA methylation plays an important role in eQTL regulation of gene expression in tumors and in benign samples. These results may guide functional studies that characterize mechanisms of genetic regulation.

## Methods

### Study populations and sample collection

The Seattle-based Fred Hutchinson (FH) Cancer Research Center PrCa study is composed of European-American male residents of King County, Washington, who were diagnosed with PrCa either in 1993–1996 or in 2002–2005 [81,82]. A subset of patients with clinically localized disease underwent radical prostatectomy as primary treatment, provided a blood sample, and provided consent for access to primary formalin-fixed paraffin-embedded (FFPE) tumor tissue obtained at surgery. Genotype data, gene expression data, and DNA methylation data were generated on this subset of PrCa patients who had blood and tumor tissue available. The details of study and sample collection have been described elsewhere [83]. In summary, the FH study includes 395 cases with both genotype data and at least one of the other data types used in the eQTL or meQTL analysis. The ages at diagnosis ranged from 35–74 years; the distribution of Gleason sum is < = 6 (49.9%), 7 (42.8%) and 8–10 (7.3%). The FH genotype data are part of the recent PRACTICAL consortium analysis which has been deposited to dbGap phs001391.v1.p. The DNA methylation data from FH is accessible at dbGap phs001921.v1.p1. The gene expression data from FH is accessible at GEO GSE141551.

The TCGA study consists of approximately 500 primary PrCa cases diagnosed in 2000–2013, mostly of European ancestry [84]. Fresh frozen prostate tumor samples were obtained by extensive pathologic, analytical, and QC review. Images of frozen tissue were evaluated by multiple expert genitourinary pathologists, and cases were excluded if no tumor cells were identifiable in the sample or if there was evidence of significant RNA degradation. The ages at diagnosis for the TCGA patients ranged from 41–78 years. Gleason scores were < = 6 (9.1%), 7 (49.9%), and 8–10 (41.0%). All TCGA data were downloaded from https://portal.gdc.cancer.gov.

The Mayo study acquired adjacent histologically benign prostate tissue from an archived collection of fresh frozen material obtained from 471 PrCa patients, the majority of whom underwent radical prostatectomy and a few who had cystoprostatectomy at the Mayo Clinic [36]. Hematoxylin and eosin (H&E) slides were prepared from each adjacent benign tissue samples to make sure all were free of prostate adenocarcinoma.

### Genotype data collection and processing

For the FH dataset, germline DNA samples (N = 395) were genotyped using two custom Illumina arrays: 1) iCOGS, with 211,000 SNPs; or 2) OncoArray-500K BeadChip with 533,000 SNPs. We applied the following QC on the SNP data: (i) excluded SNPs with call rates <95%; (ii) excluded SNPs that deviated from Hardy-Weinberg Equilibrium (HWE) at P<10^−7^; (iii) excluded SNPs for which the genotypes were discrepant in more than 2% of duplicate samples. A total of 201,598 SNPs passed the QC criteria for iCOGS data. For OncoArray data, 489,974 SNPs remained for analysis after QC. Germline DNA genotypes for TCGA samples (N = 495) were obtained from the TCGA data portal. Genotypes for 906,000 SNPs were assessed using the Affymetrix SNP 6.0 array. Confidence score was computed at each SNP, ranging from 0 (most confident) to 1 (least Confident). Genotypes with score less than 0.1 are considered to be highly confident (Broad institute, BIRDSUITE software), and 898,000 SNPs were retained in the study. For the Mayo dataset, germline DNA samples (N = 471) were genotyped using the Illumina Infinium 2.5M bead array and genotypic data were downloaded from dbGaP under accession code phs000985.v1.p1. SNPs were excluded if (i) call rate<95%; (ii) HWE<10^−5^; (iii) MAF<1%. A total of 1,541,380 SNPs were included after QC.

The three datasets utilized different microarray platforms for genotyping and none of them had all 147 PrCa risk variants genotyped. We thus imputed those missing SNPs for each dataset based on the 1000 Genomes Project. Pre-phasing was conducted using SHAPEIT [85]; IMPUTE2 was then used on the phased data to perform imputation [86]. The reference panel used was the 1000 Genomes Phase 3 release, downloaded from the IMPUTE2 website: https://mathgen.stats.ox.ac.uk/impute/1000GP_Phase3.html. After imputation, we used the following criteria to select SNPs from the three datasets: (i) imputation confidence score, INFO ≥ 0.3; and (ii) HWE p-value>1 × 10^−6^. After imputation, all 147 PrCa risk SNPs passed quality control. The top three principal components (PC) of the genome-wide genotype data were used as adjustment covariates in eQTL and meQTL analyses.

### Gene expression data collection and processing

For the FH cohort, the Human HT-12 v4 BeadChip (Illumina) was used for mRNA expression profiling of primary tumor tissues (N = 355). Expression levels for 29,377 transcripts were determined. Among those, 3326 poor quality probes (i.e., probes that matched to repeat sequences, intergenic or intronic regions, or are unlikely to provide specific signal for any transcripts) were removed from the analysis. For those genes with more than one transcript measured (n = 4601), the mean transcript level for each gene was calculated. This resulted in 18,024 genes with transcript data available for eQTL analysis. For the TCGA data, gene expression profiles (N = 492) were obtained from the TCGA data portal. The RNASeq data were generated on the Illumina HiSeq platform. The raw count data for 19,078 genes were converted to Reads Per Kilobase of gene per Million (RPKM) values. For the Mayo study, the RNASeq data were generated using the Illumina HiSeq 2000 platform, and included raw transcript counts for 17,233 genes. These data were downloaded from dbGaP under accession code phs000985.v1.p1. The raw counts data were transformed to RPKM values. In the downloaded Mayo RNA-seq data, RefSeq was originally used as the gene annotation. The gene names were in the Mayo data were converted to the GENCODE annotation, which was used in the TCGA data, in order to make the two datasets comparable in eQTL analysis. For each dataset, gene expression levels for each sample were quantile normalized using the R package *preprocessCore* [87]. For eQTL-mapping, expression levels of each gene were transformed to the quantiles of the standard normal distribution.

### DNA methylation data collection and processing

Tumor DNA was bisulfite converted, and methylome-wide CpG methylation levels were measured using the Infinium Human Methylation450 BeadChip. Background-corrected methylated (M) and unmethylated (U) summary probe intensities were measured for each CpG site, and beta values (M/(M+U)) were used in the meQTL analysis. In the FH study, tumor DNA samples (N = 377) were profiled for 485,577 CpG sites. The following QC steps were applied: (i) The R package *Minfi* was used to remove probes with a non-detection (p-value) greater than 0.05 and filter out non-CpG-targeting probes (Probe ID prefix = “ch”); and, (ii) cross-hybridizing probes and probes with any SNP within 10 bp of the CpG site or single base extension were removed [88,89,90]. Finally, for each sample 353,245 probes remained for meQTL analysis. The methylation data were normalized using the subset-quantile within array normalization (SWAN) [91], and batch effects were removed using ComBat [92]. For the TCGA study, the tumor methylation data measured on 485,577 CpG sites (N = 495) were obtained from the TCGA data portal. Probes with a non-detection (p-value) greater than 0.05 were removed by TCGA. We further applied the above filter (ii) on these data, and for each sample 396,065 probes remained for meQTL analysis.

### Identification of eQTLs

The eQTLs in the Mayo dataset were detected using the linear model, gene expression ~ SNP + top 10 PCs + age + top 15 PEER factors, to regress gene expression of a regulated gene (eGene) on a SNP, adjusting for other covariates. To remove the effect of population structure on gene expression, we used *smartpca* in the EIGENSOFT program to perform principal component (PC) analysis [93], and selected the top three PCs from genome-wide genotype data as covariates. To remove the hidden batch effects and other potential confounders in the gene expression data, we also used the Probabilistic Estimation of Expression Residuals (PEER) method to select the first 15 PEER factors as covariates [94].

The eQTLs in tumor tissue data were detected using the linear model: gene expression ~ SNP + top 10 PCs + age + top 15 PEER factors + age at diagnosis + pathologic stage + Copy Number Alterations (CNA). For these data, in addition to the above adjustment covariates used for analysis of data from adjacent-benign tissues, we also adjusted for clinical variables such as age at diagnosis and pathologic stage. We also adjusted for copy number alterations (CNA) of the eGene because somatic copy number changes can substantially affect transcript levels in tumors. For TCGA data, the CNA data were obtained from the TCGA data portal. For the FH dataset, 355 cases had both germline genotype and tumor gene expression data. Among these 355 cases, 337 also had tumor DNA methylation data available. We used the R package *ChAMP* to call CNA on the methylation data [95]; twenty additional adjacent-benign tissue samples were used as the control group to call CNA. The R package *qvalue* was used to adjust for multiple comparisons [96], and SNPs with a q-value <0.05 were defined as eQTLs. *cis*-eQTLs were defined if the SNP was within 1 Mb from the eGene region (from the first transcription start site (TSS) to the last transcription end site (TES) of an eGene).

### Identification of meQTLs

The meQTLs were detected in the FH and TCGA datasets using the linear model CpG methylation ~ SNP + top 10 PCs of genotype + top 15 PCs of methylation + age at diagnosis + pathologic stage, adjusting for covariates as follows. To remove the effect of population structure on DNA methylation, we used *smartpca* in the EIGENSOFT program to perform PC analysis [93], and selected the top three PCs of genome-wide genotype data as covariates. To remove the hidden batch effects and other confounders in the tumor methylation data, we picked the first 15 PCs of methylation data as covariates. To remove the potential effects of clinical status on DNA methylation, age at diagnosis and pathologic stage were included as additional covariates. The R package *qvalue* was used to adjust for multiple comparisons, and SNPs with a q-value <0.05 were defined as meQTLs. *cis*-meQTLs were defined if the SNP was within 1 Mb from the CpG site. All genomic data were aligned to chromosome positions from the human reference genome GRCh37/hg19.

### Analyses to explain the absence of eQTLs in tumors compared benign samples

The R/Bioconductor package *edgeR* was used for differential gene expression analysis on the 50 TCGA prostate tumors and 50 matched adjacent histologically benign samples [97]. The RNA-seq data contained counts of sequence reads aligned to 60,000 transcripts, of which 52,000 transcripts were included with at least 1 count per million (CPM) in at least two samples. The counts data were normalized using the trimmed mean of M-values (TMM) method, and differential transcripts were using the R/Bioconductor package *limma* [98].

The TCGA-PRAD level 3 somatic mutation calls for 50 tumor samples were used to compare mutation frequencies in the neighborhood of the two groups of eQTLs. For each eQTL, the number of somatic mutations within 1 Mb distance was counted for each tumor sample and the mutation frequency was computed. The TCGA-PRAD level 3 copy number segmented data for 50 tumor samples were used to compute the fraction of copy number alterations among the tumor samples for the two groups of eGenes. Copy number alterations were defined as a copy number greater than 2.5 or less than 1.5. The TCGA-PRAD level 3 DNA methylation beta values were used to compare the number of DMP and DMR between 50 TCGA tumor samples and 50 TCGA benign samples in the two groups of eGenes. For each eGene we identified the associated probes in the gene, beta values for each probe in the tumor sample group and the benign sample group were compared using the R package *geepack* [99], and the probe with FWER less than 0.05 was defined as a DMP. The methylation data were also used to compare the percentage of genes within significant meQTL-CpG pairs. For each eGene we identified the paired SNP and associated CpG probes, and checked if there were any SNP-CpG probes included in the meQTL result.

### Causal analysis of eQTL, DNA methylation, and gene expression

PrCa risk SNPs that were identified to be both eQTLs and meQTLs were analyzed for causal relationships between associated CpG methylation and gene expression. Mendelian randomization (MR) analysis was conducted by using risk SNPs as instrumental variables, testing causality by regression gene expression on predicted methylation based on genotypes using the two-stage least squares method [54]. This method assumes there is no direct effect from the genotype to gene expression, which can be problematic for this context because there are often multiple adjacent CpGs mediating the genetic effect, any single SNP-CpG-gene expression triplet may present a partial mediation. Moreover, when risk SNPs merely independently affect gene expression and CpG methylation, MR will erroneously detect causality as shown in Fig 5. To effectively distinguish the three possible relationships (Fig 7): namely the mediation relationship of CpG methylation in genetic regulation of gene expression (the SME relationship), risk SNPs independently affecting CpG methylation and gene expression, or there is reverse causation from gene expression to methylation (SEM relationship), a modified *CIT* mediation test is proposed [55], accounting for the challenge that there are typically multiple CpGs, each of which partially mediates the genetic effect. One advantage of this method over MR is that, with modification, it can test for direction of causality by switching the order of the intermediary and the outcome.

For ease of notation, suppose data contain a genotype (*G*), two correlated CpG methylation probes (*M*_*1*_ and *M*_*2*_), and a gene transcript (*Y*). The data generating models are: *M*_1_ = *α*_1_ *G* + *ε*_1_; *M*_2_ = *α*_2_*G* + *ε*_2_; *Y* = *γ*_1_*M*_1_ + *γ*_2_*M*_2_ + *ε*_3_. In this scenario, *M*_*1*_ and *M*_*2*_ together mediates the genetic effect on *Y*. If *CIT* was used to test *G*→*M*_*1*_→*Y*, three regression models will be fit: 1) E[*Y*] = β_1_*G*; 2) E[*M*_*1*_] = β_2_*G* + β_3_*Y*; 3) E[*Y*] = β_4_*G*+β_5_*M*_*1*_. The four-component test for *G*→*M*_*1*_→*Y* includes 1) β_1_ ≠ 0; 2) β_2_ ≠ 0; 3) β_5_ ≠ 0; 4) β_4_ = 0. If *CIT* is used to test *G*→*Y*→ *M*_*1*_, three regression models will be fit: 1) E[*M*_*1*_] = β_1_*G; 2) E[*Y*] = β_2_*G + β_3_*M_*1*_; 3) E[*M*_*1*_] = β_4_*G+β_5_*Y. The four-component test for *G*→*Y*→ *M*_*1*_ includes 1) β_1_* ≠ 0; 2) β_2_* ≠ 0; 3) β_5_* ≠ 0; 4) β_4_* = 0. One can show that in this scenario, *CIT* will result in significance in both directions, *G*→*M*_*1*_→*Y* and *G*→*Y*→ *M*_*1*_, a phenomenon observed previously for deciphering the direction of causality between DNA methylation and gene expression [90].

When *CIT* yielded significant p-values for both the SME and SEM relationships for many of triplets for the eQTL-eGene pair, three criteria are added to distinguish SME from SEM. First, the SME p-values should be generally smaller than the SEM p-values, as the true model should yield a smaller p-value. Second, when there is a single CpG showing significance in testing SME and SEM, the proportion of genetic association with gene expression explained by CpG methylation (for example, β_4_/ β_1_ in the models above) should be greater than the genetic association with CpG methylation explained by gene expression, both proportions between 0 and 1 (β_4_ and β_1_ having the same sign). The proportion of genetic association explained by a candidate intermediary was computed as the ratio of the genetic association without adjusting for the intermediary (fitting E[*Y*|*G*], for example) and the genetic association adjusting for the intermediary (fitting E[*Y*|*M*,*G*], for example). Third, when there are multiple adjacent CpGs showing evidence of mediation, adding multiple CpGs in the mediation model (for example, E[*Y*] = β_4_*G*+β_5_*M*+β_6_*M*_*2*_) should explain a greater proportion of the genetic effect on gene expression than any single CpG. We found in both FH and TCGA data that the third criterion often can effectively distinguish SME and SEM.

## Supporting information

S1 Fig All tumor-specific eQTL associations that were identified in one of tumor sets but not in the benign set. (PPTX)

S2 Fig Two examples of loss of or weakened eQTL association in tumor samples when compared to the tumor-adjacent benign samples. The top panels show rs4430796 and *HNF1B* in three datasets, and differential methylation at cg14694075 (a CpG site in the gene body enhancer) between tumors and adjacent benign. The bottom panels show rs547171081 and MADD, and differential methylation at cg04000940 (a CpG site in 3’ UTR and in a DNase 1 hypersensitive site) between tumors and adjacent benign.
(PPTX)

S1 Table Bioinformatic annotation of 147 prostate cancer risk SNPs. (XLSX)

S2 Table Results of eQTL and meQTL mapping for 147 prostate cancer risk SNPs among the three datasets (FH, TCGA, Mayo). (XLSX)

S3 Table Results of Ingenuity pathway analysis for eGenes corresponding to the eQTLs among 147 prostate cancer risk SNPs in the Mayo data. (XLSX)

S4 Table Results of mediation analyses for triplets of eQTL-CpG methylation-eGenes for prostate cancer risk SNPs which were both eQTL and meQTL (FH and TCGA data). (XLSX)

## References

[1] Siegel RL, Miller KD, Jemal A. Cancer statistics. CA Cancer J. Clin. 2008; 66: 7–30. 10.3322/caac.21332 26742998

[2] Cuzick J, Thorat MA, Andriole G, Brawley OW, Brown PH, Culig Z, et al Prevention and early detection of prostate cancer. Lancet Oncol. 2014; 15: e484–492. 10.1016/S1470-2045(14)70211-6 25281467 PMC4203149

[3] Stanford JL, Ostrander EA. Familial prostate cancer. Epidemiol. Rev. 2011; 23: 19–23. 10.1093/oxfordjournals.epirev.a000789 11588848

[4] Ghadirian P, Howe GR, Hislop TG, Maisonneuve P. Family history of prostate cancer: a multi-center case-control study in Canada. Int. J. Cancer. 1997; 70: 679–681. 10.1002/(sici)1097-0215(19970317)70:6<679::aid-ijc9>3.0.co;2-s 9096649

[5] Grönberg H, Damber L, Damber JE. Familial prostate cancer in Sweden: a nationwide register cohort study. Cancer. 1996; 77: 138–143. 10.1002/(SICI)1097-0142(19960101)77:1<138::AID-CNCR23>3.0.CO;2-5 8630920

[6] Matikaine MP, Pukkala E, Schleutker J, Tammela TL, Koivisto P, Sankila R, et al Relatives of prostate cancer patients have an increased risk of prostate and stomach cancers: a population-based, cancer registry study in Finland. Cancer Causes Control. 2001; 12: 223–230. 10.1023/a:1011283123610 11405327

[7] Lichtenstein P, Holm NV, Verkasalo PK, Lliadou A, Kaprio J, Koskenvuo M, et al Environmental and heritable factors in the causation of cancer-—analyses of cohorts of twins from Sweden, Denmark, and Finland. N Engl J Med. 2000; 343: 78–85. 10.1056/NEJM200007133430201 10891514

[8] Hjelmborg JB, Scheike T, Holst K, Skytthe A, Penney KL, Graff RE, et al The heritability of prostate cancer in the Nordic twin study of cancer. Cancer Epidemiol Biomarkers Prev. 2014; 23: 2303–2310. 10.1158/1055-9965.EPI-13-0568 24812039 PMC4221420

[9] Benafif S, Kote-Jarai Z, Eeles RA, PRACTICAL Consortium. A review of prostate cancer genome-wide association studies (GWAS). Cancer Epidemiol Biomarkers Prev. 2018; 27: 845–857. 10.1158/1055-9965.EPI-16-1046 29348298 PMC6051932

[10] Eeles RA, Olama AA, Benlloch S, Saunders EJ, Leongamornlert DA, Tymrakiewicz M, et al Identification of 23 new prostate cancer susceptibility loci using the iCOGS custom genotyping array. Nat. Genet. 2013; 45: 385–391. 10.1038/ng.2560 23535732 PMC3832790

[11] Al Olama AA, Kote-Jarai Z, Berndt SI, Conti DV, Schumacher F, Han Y, et al A meta-analysis of 87,040 individuals identifies 23 new susceptibility loci for prostate cancer. Nat. Genet. 2014; 46: 1103–1109. 10.1038/ng.3094 25217961 PMC4383163

[12] Al Olama AA, Kote-Jarai Z, Giles GG, Guy M, Morrison J, Severi G, et al Multiple loci on 8q24 associated with prostate cancer susceptibility. Nat. Genet. 2019; 41: 1058–1060. 10.1038/ng.452 19767752

[13] Amundadottir LT, Sulem P, Gudmundsson J, Helgason A, Baker A, et al A common variant associated with prostate cancer in European and African populations. Nat. Genet. 2006; 38: 652–658. 10.1038/ng1808 16682969

[14] Eeles RA, Kote-Jarai Z, Al Olama AA, Giles GG, Guy M, Severi G, et al Identification of seven new prostate cancer susceptibility loci through a genome-wide association study. Nat. Genet. 2009; 41: 1116–1121. 10.1038/ng.450 19767753 PMC2846760

[15] Eeles RA, Kote-Jarai Z, Giles GG, Olama AA, Guy M, Jugurnauth SK, et al Multiple newly identified loci associated with prostate cancer susceptibility. Nat. Genet. 2008; 40: 316–321. 10.1038/ng.90 18264097

[16] Gudmundsson J, Sulem P, Gudbjartsson DF, Blondal T, Gylfason A, Agnarsson BA, et al Genome-wide association and replication studies identify four variants associated with prostate cancer susceptibility. Nat. Genet. 2009; 41: 1122–1126. 10.1038/ng.448 19767754 PMC3562712

[17] Gudmundsson J, Sulem P, Manolescu A, Amundadottir LT, Gudbjartsson D, Helgason A, et al Genome-wide association study identifies a second prostate cancer susceptibility variant at 8q24. Nat. Genet. 2007; 39: 631–637. 10.1038/ng1999 17401366

[18] Gudmundsson J, Sulem P, Rafnar T, Bergthorsson JT, Manolescu A, Gudbjartsson D, et al Common sequence variants on 2p15 and Xp11.22 confer susceptibility to prostate cancer. Nat. Genet. 2008; 40: 281–283. 10.1038/ng.89 18264098 PMC3598012

[19] Gudmundsson J, Sulem P, Steinthorsdottir V, Bergthorsson JT, Thorleifsson G, Manolescu A, et al Two variants on chromosome 17 confer prostate cancer risk, and the one in *TCF2* protects against type 2 diabetes. Nat. Genet. 2007, 39: 977–983. 10.1038/ng2062 17603485

[20] Haiman CA, Chen GK, Blot WJ, Strom SS, Berndt SI, Kittles RA, et al Genome-wide association study of prostate cancer in men of African ancestry identifies a susceptibility locus at 17q21. Nat. Genet. 2011; 43: 570–573. 10.1038/ng.839 21602798 PMC3102788

[21] Kote-Jarai Z, Olama AA, Giles GG, Severi G, Schleutker J, Weischer M, et al Seven prostate cancer susceptibility loci identified by a multi-stage genome-wide association study. Nat. Genet. 2011; 43: 785–791. 10.1038/ng.882 21743467 PMC3396006

[22] Schumacher FR, Berndt SI, Siddiq A, Jacobs KB, Wang Z, Lindstrom S, et al Genome-wide association study identifies new prostate cancer susceptibility loci. Hum. Mol. Genet. 2011; 20: 3867–3875. 10.1093/hmg/ddr295 21743057 PMC3168287

[23] Sun J, Zheng SL, Wiklund F, Isaacs SD, Purcell LD, Gao Z, et al Evidence for two independent prostate cancer risk-associated loci in the HNF1B gene at 17q12. Nat. Genet. 2008; 40: 1153–1155. 10.1038/ng.214 18758462 PMC3188432

[24] Takata R, Akamatsu S, Kubo M, Takahashi A, Hosono N, Kawaguchi T, et al Genome-wide association study identifies five new susceptibility loci for prostate cancer in the Japanese population. Nat. Genet. 2010; 42: 751–754. 10.1038/ng.635 20676098

[25] Thomas G, Jacobs KB, Yeager M, Kraft P, Wacholder S, Orr N, et al Multiple loci identified in a genome-wide association study of prostate cancer. Nat. Genet. 2008; 40: 310–315. 10.1038/ng.91 18264096

[26] Yeager M, Orr N, Hayes RB, Jacobs KB, Kraft P, Wacholder S, et al Genome-wide association study of prostate cancer identifies a second risk locus at 8q24. Nat. Genet. 2007; 39: 645–649. 10.1038/ng2022 17401363

[27] Duggan D, Zheng SL, Knowlton M, Benitez D, Dimitrov L, Wiklund F, et al Two genome-wide association studies of aggressive prostate cancer implicate putative prostate tumor suppressor gene DAB2IP. J. Natl. Cancer Inst. 2007; 99: 1836–1844. 10.1093/jnci/djm250 18073375

[28] Amin Al Olama A, Kote-Jarai Z, Schumacher FR, Wiklund F, Berndt SI, Benlloch S, et al A meta-analysis of genome-wide association studies to identify prostate cancer susceptibility loci associated with aggressive and non-aggressive disease. Hum. Mol. Genet. 2013; 22: 408–415. 10.1093/hmg/dds425 23065704 PMC3526158

[29] Schumacher FR, Olama AAA, Berndt SI, Benlloch S, Ahmed M, Saunders EJ, et al Association analyses of more than 140,000 men identify 63 new prostate cancer susceptibility loci. Nat. Genet. 2018; 50: 928–936. 10.1038/s41588-018-0142-8 29892016 PMC6568012

[30] Albert FW, Kruglyak L. The role of regulatory variation in complex traits and disease. Nat. Rev. Genet. 2015; 16: 197–212. 10.1038/nrg3891 25707927

[31] Dixon AL, Liang L, Moffatt MF, Chen W, Heath S, Wong KC, et al A genome-wide association study of global gene expression. Nat. Genet. 2007; 39: 1202–1207. 10.1038/ng2109 17873877

[32] Spielman RS, Bastone LA, Burdick JT, Morley M, Ewens WJ, Cheung VG. Common genetic variants account for differences in gene expression among ethnic groups. Nat. Genet. 2007; 39: 226–231. 10.1038/ng1955 17206142 PMC3005333

[33] Stranger BE, Nica AC, Forrest MS, Dimas A, Bird CP, Beazley C, et al Population genomics of human gene expression. Nat. Genet. 2007; 39: 1217–1224. 10.1038/ng2142 17873874 PMC2683249

[34] GTEx Consortium. The Genotype-Tissue Expression (GTEx) project. Nat. Genet. 2013; 45: 580–585. 10.1038/ng.2653 23715323 PMC4010069

[35] GTEx Consortium. Genetic effects on gene expression across human tissues. Nature. 2017; 550: 204–213. 10.1038/nature24277 29022597 PMC5776756

[36] Thibodeau SN, French AJ, McDonnell SK, Cheville J, Middha S, Tillmans L, et al Identification of candidate genes for prostate cancer-risk SNPs utilizing a normal prostate tissue eQTL data set. Nat. Commun. 2015; 6: 8653 10.1038/ncomms9653 26611117 PMC4663677

[37] Nicolae DL, Gamazon E, Zhang W, Duan S, Dolan ME, Cox NJ. Trait-associated SNPs are more likely to be eQTLs: annotations to enhance discovery from GWAS. PLoS Genetics. 2010; 6: e1000888 10.1371/journal.pgen.1000888 20369019 PMC2848547

[38] Grisanzio C, Werner L, Takeda D, Awoyemi BC, Pomerantz MM, Yamada H, et al Genetic and functional analyses implicate the NUDT11, HNF1B and SLC22A3 genes in prostate cancer pathogenesis. PNAS. 2012; 109: 11252–11257. 10.1073/pnas.1200853109 22730461 PMC3396469

[39] Penney KL, Sinnott JA, Tyekucheva S, Gerke T, Shui IM, Kraft P, et al Association of prostate cancer risk variants with gene expression in normal and tumor tissue. Cancer Epidemiol Biomarkers Prev. 2015; 24: 255–260. 10.1158/1055-9965.EPI-14-0694-T 25371445 PMC4294966

[40] Wagner JR, Busche S, Ge B, Kwan T, Pastinen T, Blanchette M. The relationship between DNA methylation, genetic and expression inter-individual variation in untransformed human fibroblasts. Genome Biol. 2014; 15: R37 10.1186/gb-2014-15-2-r37 24555846 PMC4053980

[41] Banovich NE, Lan X, McVicker G, van de Geijn B, Degner JF, Blischak JD, et al Methylation QTLs are associated with coordinated changes in transcription factor binding, histone modifications, and gene expression levels. PLoS Genet. 2014; 10: e1004663 10.1371/journal.pgen.1004663 25233095 PMC4169251

[42] Lemire M, Zaidi SH, Ban M, Ge B, Aïssi D, Germain M, et al Long-range epigenetic regulation is conferred by genetic variation located at thousands of independent loci. Nat. Commun. 2015; 6: 6326 10.1038/ncomms7326 25716334 PMC4351585

[43] Portela A, Esteller M. Epigenetic modifications and human disease. Nat. Biotechnol. 2010; 28: 1057–1068. 10.1038/nbt.1685 20944598

[44] Massie CE, Mills IG, Lynch AG. The importance of DNA methylation in prostate cancer development Identification. Journal of Steriod Biochemistry and Molecular Biology. 2017; 166: 1–15. 10.1016/j.jsbmb.2016.04.009 27117390

[45] Gibbs JR, van der Brug MP, Hernandez DG, Traynor BJ, Nalls MA, Lai SL, et al Abundant quantitative trait loci exist for DNA methylation and gene expression in human brain. PLoS Genet. 2010; 6: e1000952 10.1371/journal.pgen.1000952 20485568 PMC2869317

[46] van Eijk KR, de Jong S, Boks MP, Langeveld T, Colas F, Veldink JH, et al Genetic analysis of DNA methylation and gene expression levels in whole blood of healthy human subjects. BMC Genomics. 2012; 13: 636 10.1186/1471-2164-13-636 23157493 PMC3583143

[47] Smith AK, Kilaru V, Kocak M, Almli LM, Mercer KB, Ressler KJ, et al Methylation quantitative trait loci (meQTLs) are consistently detected across ancestry, developmental stage, and tissue type. BMC Genomics. 2014; 15: 145 10.1186/1471-2164-15-145 24555763 PMC4028873

[48] Drong AW, Nicholson G, Hedman AK, Meduri E, Grundberg E, Small KS, et al The presence of methylation quantitative trait loci indicates a direct genetic influence on the level of DNA methylation in adipose tissue. PLoS One. 2013; 8: e55923 10.1371/journal.pone.0055923 23431366 PMC3576415

[49] Quon G, Lippert C, Heckerman D, Listgarten J. Patterns of methylation heritability in a genome-wide analysis of four brain regions. Nucleic Acids Res. 2013; 41: 2095–2104. 10.1093/nar/gks1449 23303775 PMC3575819

[50] Banovich NE, Lan X, McVicker G, van de Geijn B, Degner JF, Blischak JD, et al Methylation QTLs are associated with coordinated changes in transcription factor binding, histone modifications, and gene expression levels. PLoS Genet. 2014; 10: e1004663 10.1371/journal.pgen.1004663 25233095 PMC4169251

[51] Gutierrez-Arcelus M, Lappalainen T, Montgomery SB, Buil A, Ongen H, Yurovsky A, et al Passive and active DNA methylation and the interplay with genetic variation in gene regulation. eLife. 2013; 2: e00523 10.7554/eLife.00523 23755361 PMC3673336

[52] van Eijk KR, de Jong S, Boks MP, Langeveld T, Colas F, Veldink JH, et al Genetic analysis of DNA methylation and gene expression levels in whole blood of healthy human subjects. BMC Genomics. 2012; 13: 636 10.1186/1471-2164-13-636 23157493 PMC3583143

[53] Pierce BL, Tong L, Argos M, Demanelis K, Jasmine F, Rakibuz-Zaman M, et al Co-occurring expression and methylation QTLs allow detection of common causal variants and shared biological mechanisms. Nat. Commun. 2018; 9: 804 10.1038/s41467-018-03209-9 29476079 PMC5824840

[54] Hemani G, Tilling K, Davey Smith G. Orienting the causal relationship between imprecisely measured traits using GWAS summary data. PLoS Genetics. 2017; 13: e1007081 10.1371/journal.pgen.1007081 29149188 PMC5711033

[55] Millstein J, Zhang B, Zhu J, Schadt EE. Disentangling molecular relationships with a causal inference test. BMC Genet. 2009; 10: 23 10.1186/1471-2156-10-23 19473544 PMC3224661

[56] Troyer DA, Lucia MS, de Bruïne AP, Mendez-Meza R, Baldewijns MM, Dunscomb N, et al Prostate Cancer Detected by Methylated Gene Markers in Histopathologically cancer-negative tissues from men with subsequent positive biopsies. Cancer Epidemiol Biomarkers Prev. 2009; 18(10): 2717–2722. 10.1158/1055-9965.EPI-09-0068 19755651

[57] Kosari F, Cheville JC, Ida CM, Karnes RJ, Leontovich AA, Sebo TJ, et al Shared gene expression alterations in prostate cancer and histologically benign prostate from patients with prostate cancer. American Journal of Pathology. 2012; 181(1): 34–42. 10.1016/j.ajpath.2012.03.043 22640805 PMC3388167

[58] Moller M, Strand SH, Mundbjerg K, Liang G, Gill I, Haldrup C, et al Heterogeneous patterns of DNA methylation-based field effects in histologically normal prostate tissue from cancer patients. Scientific Reports. 2017; 7: 40636 10.1038/srep40636 28084441 PMC5233981

[59] Nguyen HH, Takata R, Akamatsu S, Shigemizu D, Tsunoda T, Furihata M, et al IRX4 at 5p15 suppresses prostate cancer growth through interaction with vitamin D receptor, conferring prostate cancer susceptibility. Hum Mol Genet. 2012; 21: 2076–2085. 10.1093/hmg/dds025 22323358

[60] Xu X, Hussain WM, Vijai J, Offit K, Rubin MA, Demichelis F, et al Variants at IRX4 as prostate cancer expression quantitative trait loci. Eur J Hum Genet. 2014; 22**:** 558–563. 10.1038/ejhg.2013.195 24022300 PMC3953920

[61] Ross-Adams H, Ball S, Lawrenson K, Halim S, Russell R, Wells C, et al HNF1B variants associate with promoter methylation and regulate gene networks activated in prostate and ovarian cancer. Oncotarget. 2016; 7: 74734–74746. 10.18632/oncotarget.12543 27732966 PMC5342698

[62] Hu YL, Zhong D, Pang F, Ning QY, Zhang YY, Li G, et al HNF1B is involved in prostate cancer risk via modulating androgenic hormone effects and coordination with other genes. Genet Mol Res. 2013; 12: 1327–1335. 10.4238/2013.April.25.4 23661456

[63] Liao D. Emerging role of the EBF family of transcription factors in tumor suppression. Mol Cancer Res. 2009; 7: 1893–1901. 10.1158/1541-7786.MCR-09-0229 19996307 PMC5545892

[64] Amin Al Olama A, Dadaev T, Hazelett DJ, Li Q, Leongamornlert D, Saunders EJ, et al Multiple novel prostate cancer susceptibility signals identified by fine-mapping of known risk loci among Europeans. Hum Mol Genet. 2015; 24(19): 5589–5602. 10.1093/hmg/ddv203 26025378 PMC4572072

[65] Li Q, Stram A, Chen C, Kar S, Gayther S, Pharoah P, et al Expression QTL-based analyses reveal candidate causal genes and loci across five tumor types. Human Molecular Genetics 2014; 23: 5294–5302. 10.1093/hmg/ddu228 24907074 PMC4215106

[66] Nickerson ML, Das S, Im KM, Turan S, Berndt SI, Li H, et al TET2 binds the androgen receptor and loss is associated with prostate cancer. Oncogene 2017; 36(15): 2172–2183. 10.1038/onc.2016.376 27819678 PMC5391277

[67] Patra SK, Petra A, Zhao H, Dahiya R. DNA methyltransferase and demethylase in human prostate cancer. Mol Carcinog. 2002; 33(3): 163–171. 10.1002/mc.10033 11870882

[68] Seetharaman S, Flemyng E, Shen J, Conte MR, Ridley AJ. The RNA-binding protein LARP4 regulates cancer cell migration and invasion. Cytoskeleton (Hoboken). 2006; 73(11): 680–690. 10.1002/cm.21336 PMC5111583 27615744

[69] Bu H, Narisu N, Schlick B, Rainer J, Manke T, Schäfer G, et al Putative prostate cancer risk SNP in an androgen receptor-binding site of the melanophilin gene illustrates enrichment of risk sNPs in androgen receptor target sites. Hum. Mutat. 2016; 37(1): 52–64. 10.1002/humu.22909 26411452 PMC4715509

[70] Bodle CR, Mackie DI, Roman DL. RGS17: an emerging therapeutic target for lung and prostate cancers. Future Med Chem. 2013; 5(9): 995–1007. 10.4155/fmc.13.91 23734683 PMC3865709

[71] James MA, Lu Y, Liu Y, Vikis HG, You M. RGS17, an overexpressed gene in human lung and prostate cancer, induces tumor cell proliferation through the cyclic AMP-PKA-CREB pathway. Cancer Research. 2009; 69(5): 2018–2016. 10.1158/0008-5472.CAN-08-3589 19244110 PMC2746047

[72] Pomerantz MM, Shrestha Y, Flavin RJ, Regan MM, Penney KL, Mucci LA, et al Analysis of the 10q11 cancer risk locus implicates MSMB and NCOA4 in human prostate tumorigenesis. PLoS genetics 2010; 6: e1001204 10.1371/journal.pgen.1001204 21085629 PMC2978684

[73] Whitaker HC, Kote-Jarai Z, Ross-Adams H, Warren AY, Burge J, George A, et al The rs10993994 risk allele for prostate cancer results in clinically relevant changes in microseminoprotein-beta expression in tissue and urine. PloS One 2010; 5: e13363 10.1371/journal.pone.0013363 20967219 PMC2954177

[74] Han Y, Hazelett DJ, Wiklund F, Schumacher FR, Stram DO, Berndt SI, et al Integration of multiethnic fine-mapping and genomic annotation to prioritize candidate functional SNPs at prostate cancer susceptibility regions. Hum Mol Genet. 2015; 24(19): 5603–5618. 10.1093/hmg/ddv269 26162851 PMC4572069

[75] He XH, Li JJ, Xie YH, Tang YT, Yao GF, Qin WX, et al Altered gene expression profiles of NIH3T3 cells regulated by human lung cancer associated gene CT120. Cell Res. 2004 14(6): 487–496. 10.1038/sj.cr.7290252 15625016

[76] Zhang J, Kuang Y, Wang Y, Xu Q, Ren Q. Notch-4 silencing inhibits prostate cancer growth and EMT via the NF-κB pathway. Apoptosis 2017; 22(6): 877–884. 10.1007/s10495-017-1368-0 28374086

[77] Ongen H, Andersen CL, Bramsen JB, Oster B, Rasmussen MH, Ferreira PG, et al Putative cis-regulatory drivers in colorectal cancer. Nature. 2014; 512: 87–90. 10.1038/nature13602 25079323

[78] Drake CG. Prostate cancer as a model for tumour immunotherapy. Nat. Rev. Immunol. 2010; 10: 580–593. 10.1038/nri2817 20651745 PMC3082366

[79] Wang X, Yu J, Sreekumar A, Varambally S, Shen R, Giacherio D, et al Antibody signatures in prostate cancer. N. Engl. J. Med. 2005; 353: 1224–1235. 10.1056/NEJMoa051931 16177248

[80] Noguchi M, Koga N, Moriya F, Itoh K. Immunotherapy in prostate cancer: challenges and opportunities. Immunotherapy 2016; 8: 69–77. 10.2217/imt.15.101 26642100

[81] Agalliu I, Salinas CA, Hansten PD, Ostrander EA, Stanford JL. Statin use and risk of prostate cancer: results from a population-based epidemiologic study. Am J Epidemiol. 2008; 168: 250–260. 10.1093/aje/kwn141 18556686 PMC2585510

[82] Stanford JL, Wicklund KG, McKnight B, Daling JR, Brawer MK. Vasectomy and risk of prostate cancer. Cancer Epidemiol Biomarkers Prev. 1999; 8: 881–886. 10548316

[83] Zhao S, Geybels MS, Leonardson A, Rubicz R, Kolb S, Yan Q, et al Epigenome-wide tumor DNA methylation profiling identifies novel prognostic biomarkers of metastatic-lethal progression in men diagnosed with clinically localized prostate cancer. Clinical Cancer Research. 2017; 23: 311–319. 10.1158/1078-0432.CCR-16-0549 27358489 PMC5199634

[84] The Cancer Genome Atlas Research Network. The molecular taxonomy of primary prostate cancer. Cell. 2015; 163: 1011–1025. 10.1016/j.cell.2015.10.025 26544944 PMC4695400

[85] Delaneau O, Marchini J, Zagury J. A linear complexity phasing method for thousands of genomes. Nature Methods. 2011; 9: 179–181. 10.1038/nmeth.1785 22138821

[86] Howie BN, Donnelly P, Marchini J. A flexible and accurate genotype imputation method for the next generation of genome-wide association studies. PLoS Genetics. 2009; 5: e1000529 10.1371/journal.pgen.1000529 19543373 PMC2689936

[87] Bolstad B. preprocessCore: A collection of pre-processing functions. R package version 1.44.0, https://github.com/bmbolstad/preprocessCore. 2018.

[88] Aryee MJ, Jaffe AE, Corrada-Bravo H, Ladd-Acosta C, Feinberg AP, Hansen KD, et al Minfi: a flexible and comprehensive Bioconductor package for the analysis of Infinium DNA methylation microarrays. Bioinformatics. 2014; 30(10): 1363–1369. 10.1093/bioinformatics/btu049 24478339 PMC4016708

[89] Chen Y, Lemire M, Choufani S, Butcher DT, Grafodatskaya D, Zanke BW, et al Discovery of cross-reactive probes and polymorphic CpGs in the Illumina Infinium HumanMethylation450 microarray. Epigenetics. 2013; 8(2): 203–209. 10.4161/epi.23470 23314698 PMC3592906

[90] Hannon E, Spiers H, Viana J, Pidsley R, Burrage J, Murphy TM, et al Methylation QTLs in the developing brain and their enrichment in schizophrenia risk loci, Nature Neuroscience. 2016; 19(1): 48–54. 10.1038/nn.4182 26619357 PMC4714325

[91] Johnson WE, Li C, Rabinovic A. Adjusting batch effects in microarray expression data using empirical Bayes methods. Biostatistics. 2007; 8: 118–127. 10.1093/biostatistics/kxj037 16632515

[92] Maksimovic J, Gordon L, Oshlack A. SWAN: Subset-quantile within array normalization for Illumina Infinium HumanMethylation450 BeadChips. Genome Biology. 2012; 13: R44 10.1186/gb-2012-13-6-r44 22703947 PMC3446316

[93] Price AL, Patterson NJ, Plenge RM, Weinblatt ME, Shadick NA, Reich D. Principal components analysis corrects for stratification in genome-wide association studies. Nat. Genet. 2006; 38: 904–909. 10.1038/ng1847 16862161

[94] Stegle O, Parts L, Durbin R, Winn J. A Bayesian framework to account for complex non-genetic factors in gene expression levels greatly increases power in eQTL studies. PLoS Computational Biology. 2010; 6: e1000770 10.1371/journal.pcbi.1000770 20463871 PMC2865505

[95] Tian Y, Morris TJ, Webster AP, Yang Z, Beck S, Feber A, et al ChAMP: updated methylation analysis pipeline for Illumina BeadChips, Bioinformatics. 2017; 33(24): 3982–3984. 10.1093/bioinformatics/btx513 28961746 PMC5860089

[96] Storey JD, Bass AJ, Dabney A, Robinson D, Warnes G. qvalue: Q-value estimation for false discovery rate control. R package version 2.14.1, http://github.com/jdstorey/qvalue. 2019.

[97] Robinson MD, McCarthy DJ, Smyth GK. edgeR: a Bioconductor package for differential expression analysis of digital gene expression data. Bioinformatics. 2010; 26(1): 139–140. 10.1093/bioinformatics/btp616 19910308 PMC2796818

[98] Ritchie ME, Phipson B, Wu D, Hu Y, Law CW, Shi W, et al Limma powers differential expression analyses for RNA-sequencing and microarray studies. Nucleic Acids Research. 2015; 43(7): e47 10.1093/nar/gkv007 25605792 PMC4402510

[99] Højsgaard S, Halekoh U, Yan J. The R package geepack for generalized estimating equations. Journal of Statistical Software. 2006; 15(2): 1–11.

