# Supplementary figures and images for: DNA methylation and cis-regulation of gene expression by prostate cancer risk SNPs
Source: PLoS Genet. 2020 Mar 30;16(3):e1008667. doi: 10.1371/journal.pgen.1008667 (PMC7145271; doi:10.1371/journal.pgen.1008667)

## Slide 1
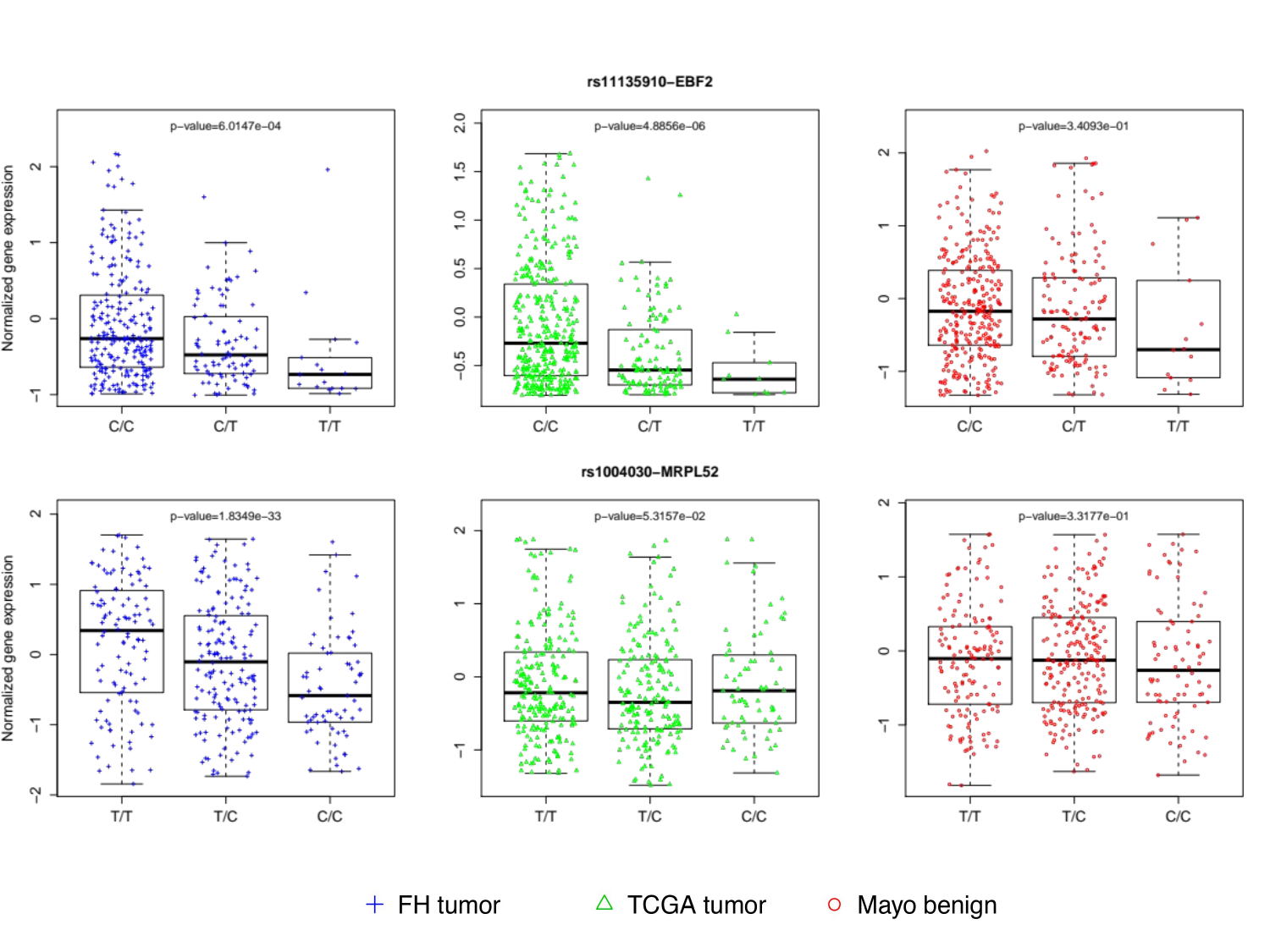

## Slide 2
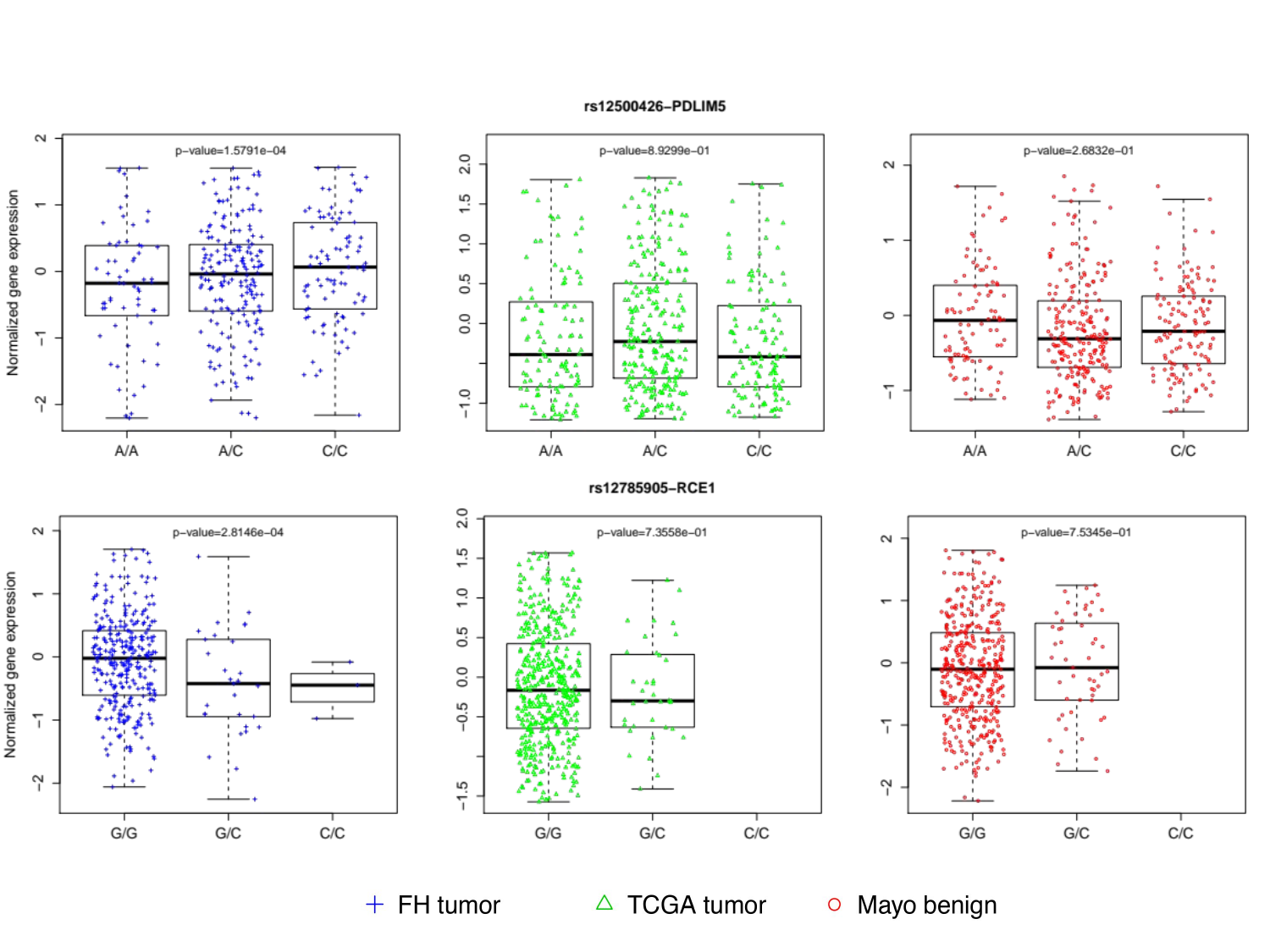

## Slide 3
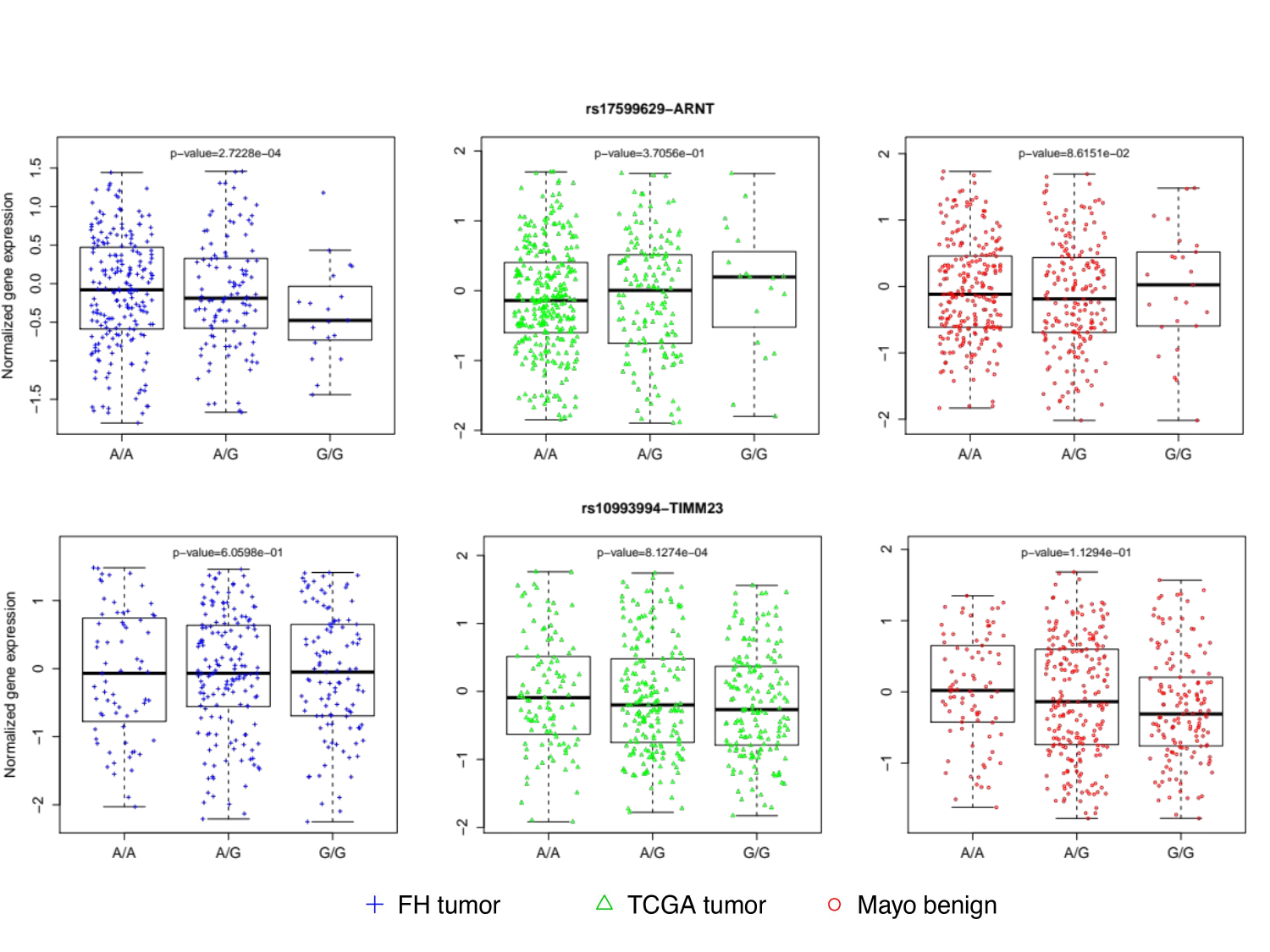

## Slide 4
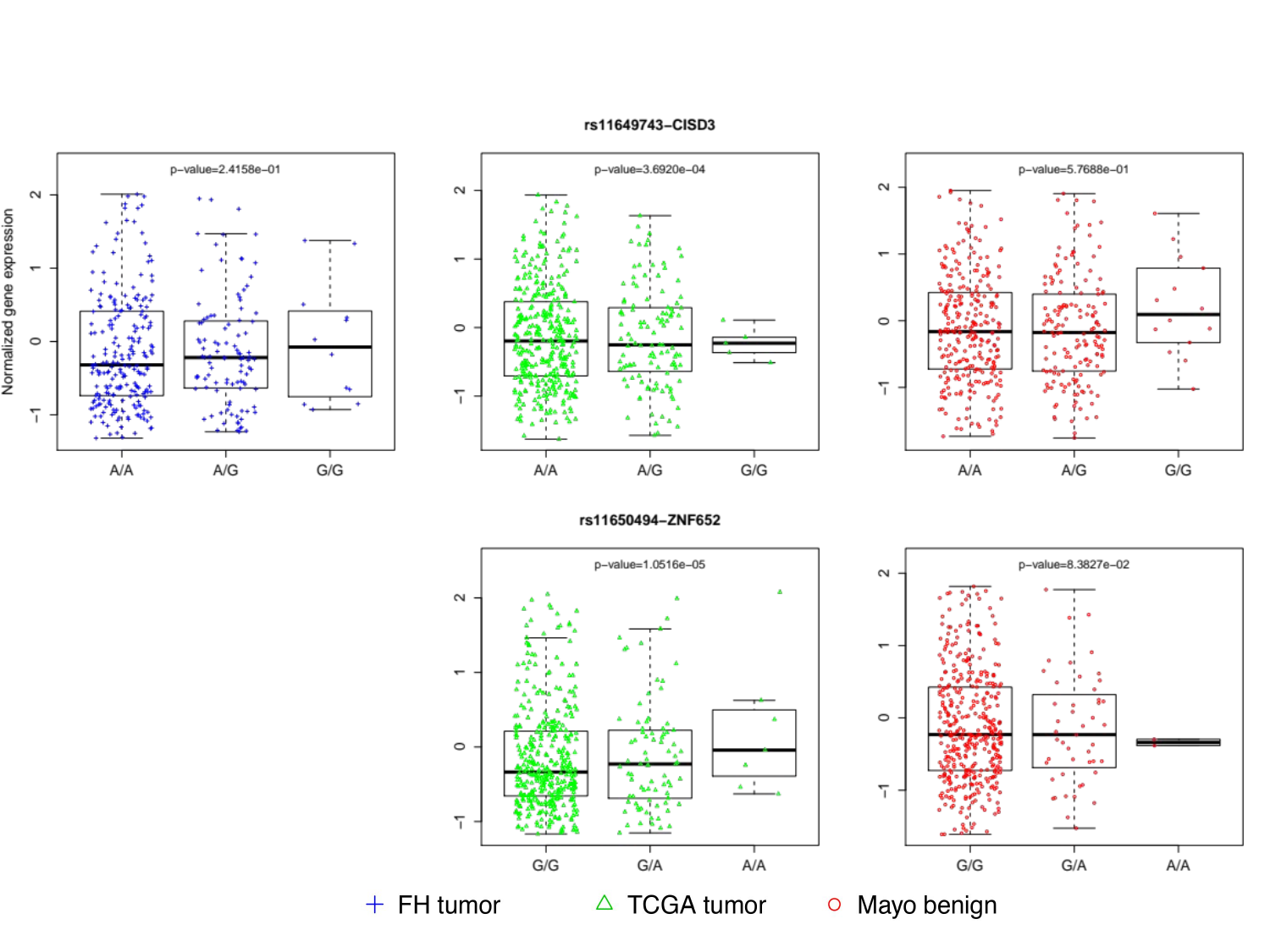

## Slide 5
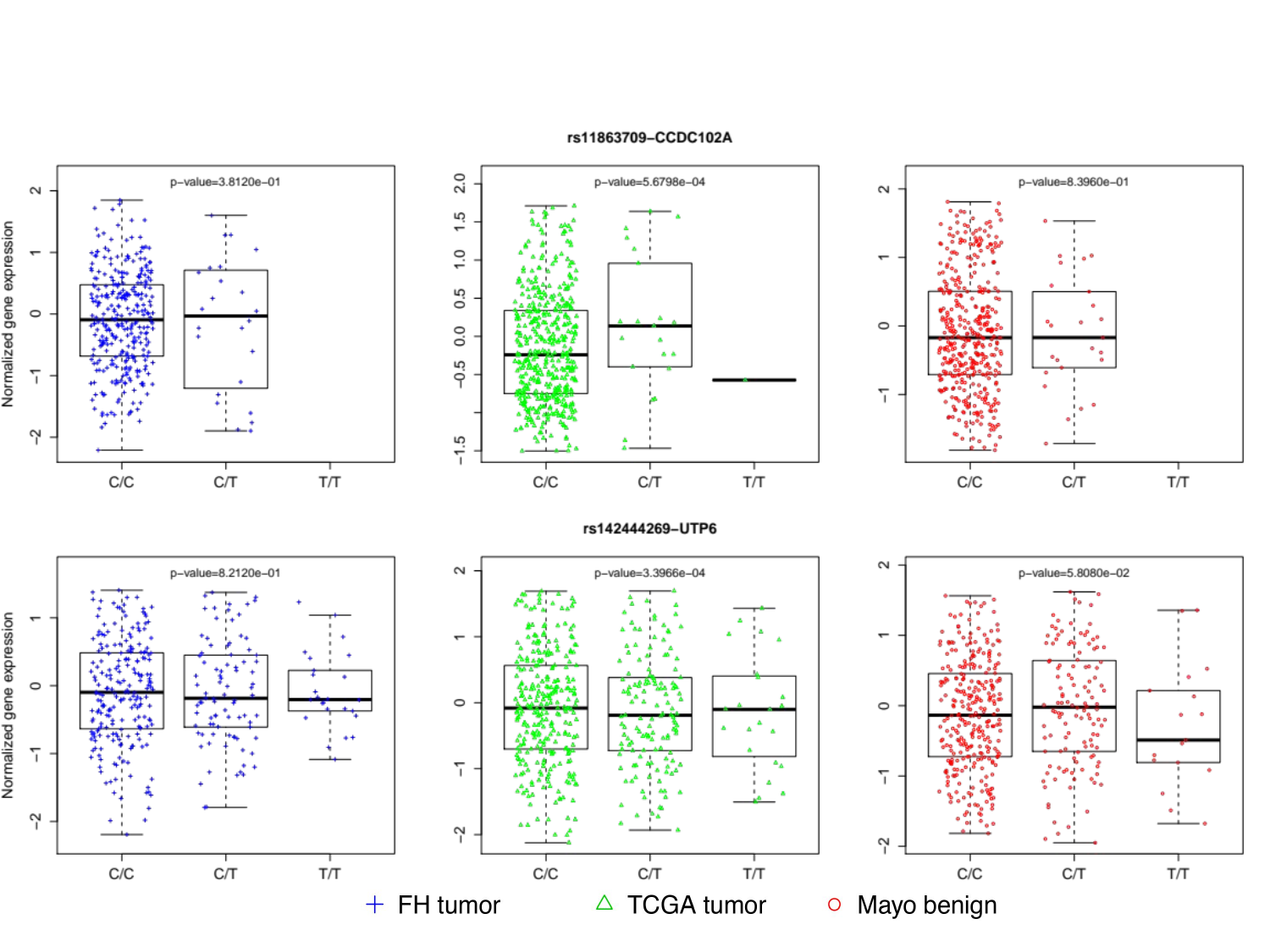

## Slide 6
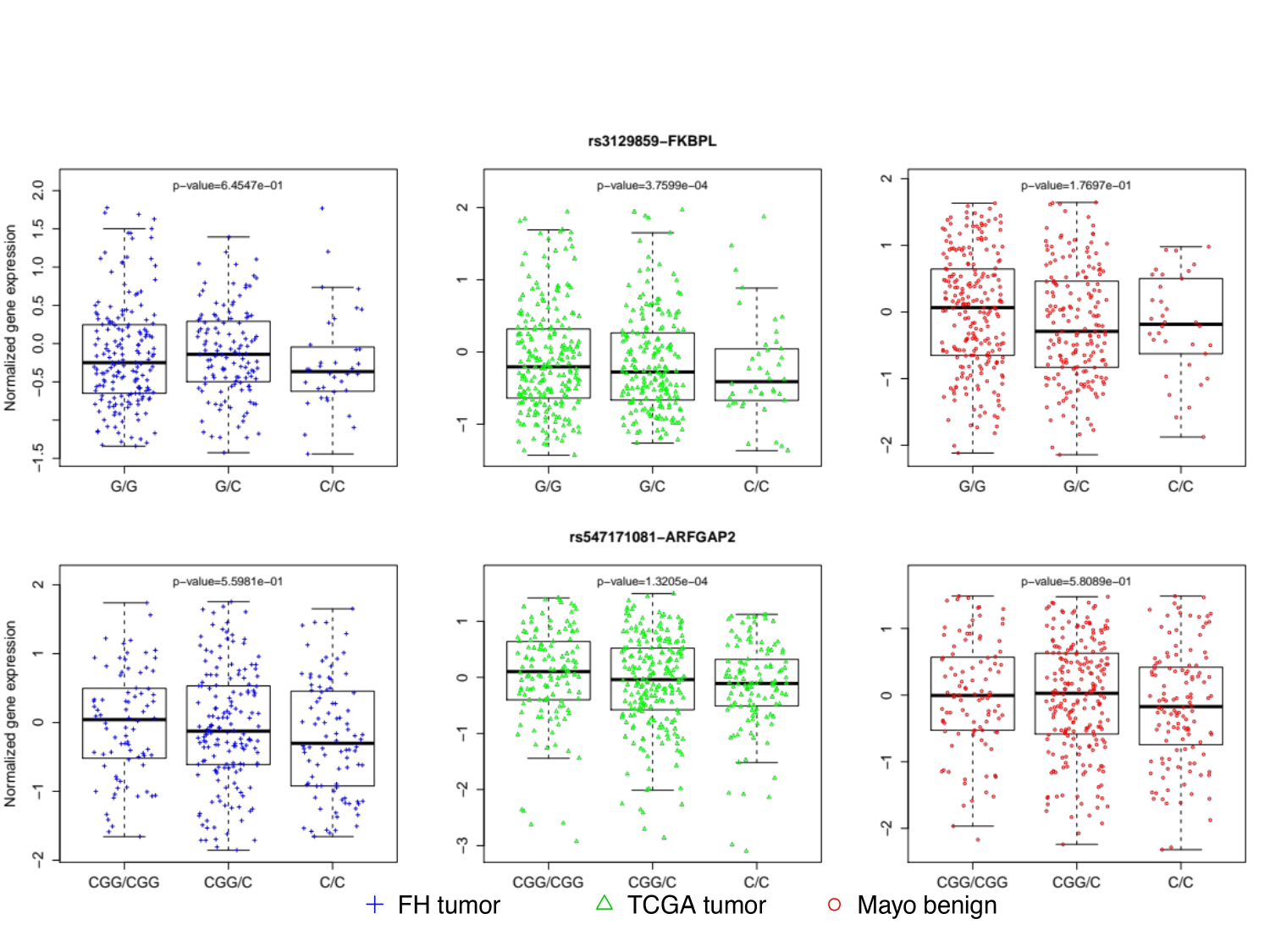

## Slide 7
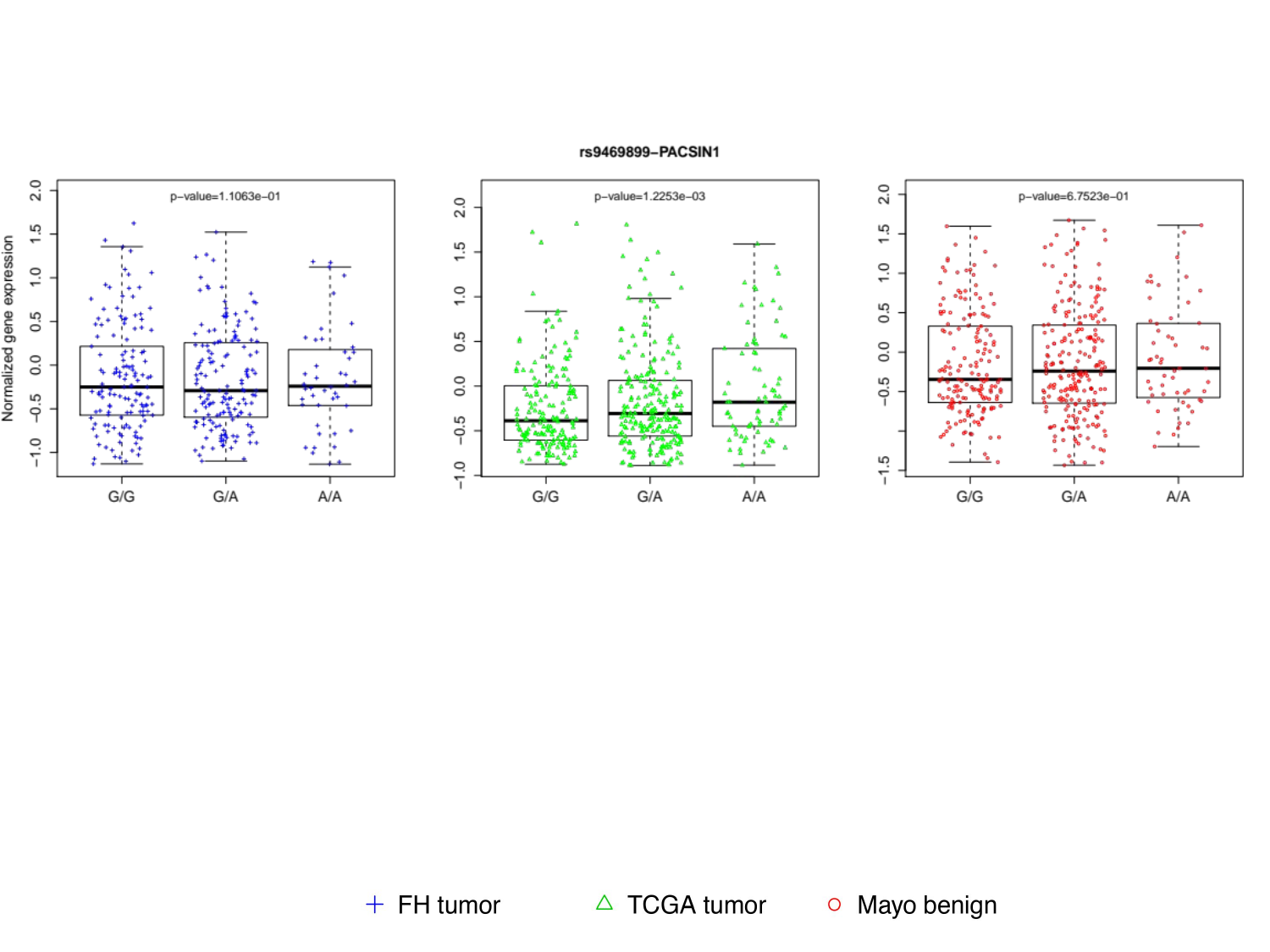

Supplement: S1 Fig — (PPTX) [file pgen.1008667.s001.pptx]
