# Supplementary material for: DNA methylation and cis-regulation of gene expression by prostate cancer risk SNPs
Source: PLoS Genet. 2020 Mar 30;16(3):e1008667. doi: 10.1371/journal.pgen.1008667 (PMC7145271; doi:10.1371/journal.pgen.1008667)
Supplement: S2 Fig — The top panels show rs4430796 and HNF1B in three datasets, and differential methylation at cg14694075 (a CpG site in the gene body enhancer) between tumors and adjacent benign. The bottom panels show rs547171081 and MADD, and differential methylation at cg04000940 (a CpG site in 3’ UTR and in a DNase 1 hypersensitive site) between tumors and adjacent benign. (PPTX) [file pgen.1008667.s002.pptx]

## Slide 1
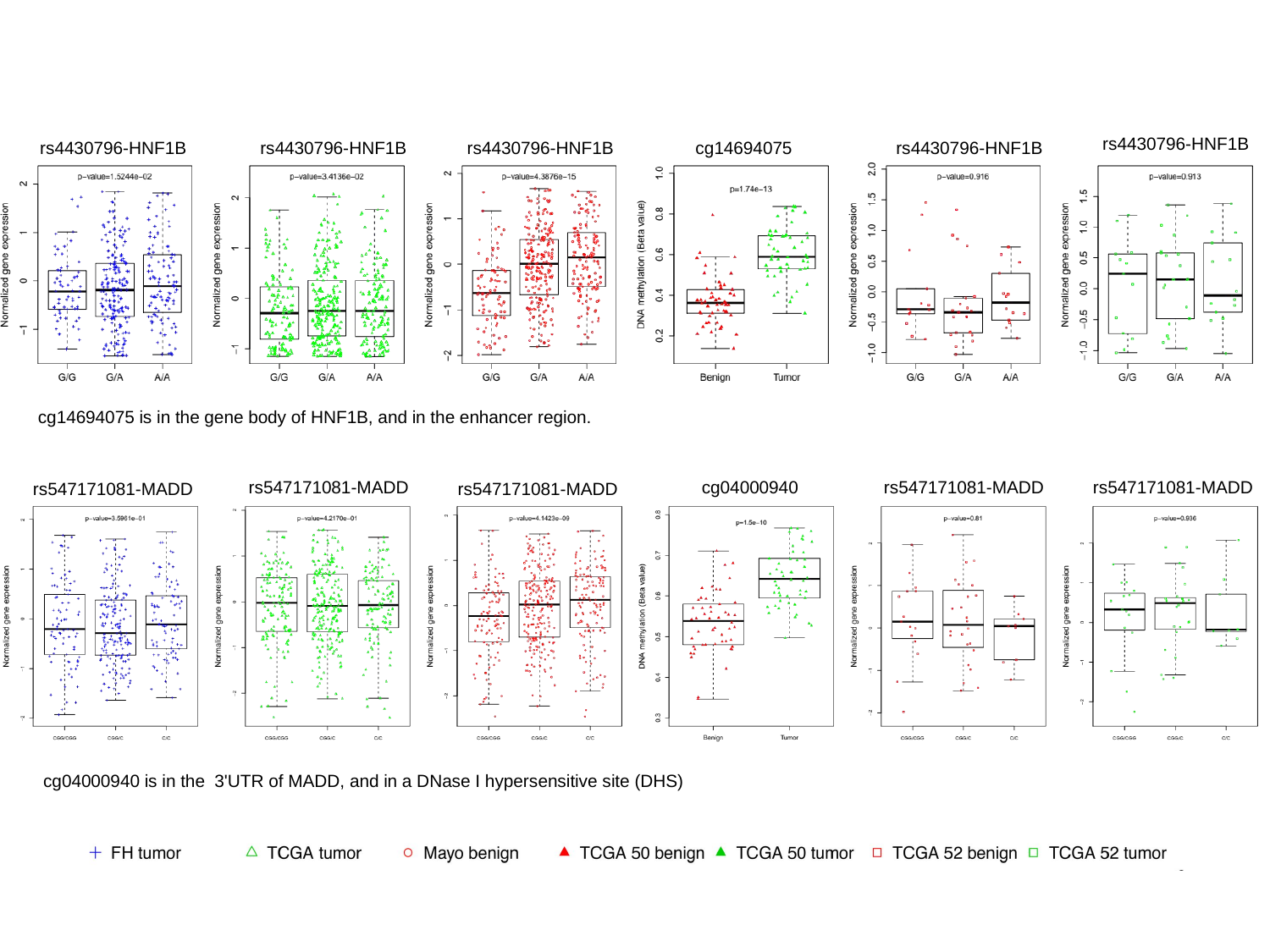

rs4430796-HNF1B
rs4430796-HNF1B
rs4430796-HNF1B
rs4430796-HNF1B
rs4430796-HNF1B
cg14694075
cg14694075 is in the gene body of HNF1B, and in the enhancer region.
rs547171081-MADD
cg04000940
rs547171081-MADD
rs547171081-MADD
rs547171081-MADD
rs547171081-MADD
cg04000940 is in the 3'UTR of MADD, and in a DNase I hypersensitive site (DHS)
